# The IQGAP scaffolds: Critical nodes bridging receptor activation to cellular signaling

**DOI:** 10.1083/jcb.202205062

**Published:** 2023-04-18

**Authors:** Louise Thines, Francis J. Roushar, Andrew C. Hedman, David B. Sacks

**Affiliations:** 1 Department of Laboratory Medicine, National Institutes of Health Clinical Center, Bethesda, MD, USA

## Abstract

The scaffold protein IQGAP1 assembles multiprotein signaling complexes to influence biological functions. Cell surface receptors, particularly receptor tyrosine kinases and G-protein coupled receptors, are common IQGAP1 binding partners. Interactions with IQGAP1 modulate receptor expression, activation, and/or trafficking. Moreover, IQGAP1 couples extracellular stimuli to intracellular outcomes via scaffolding of signaling proteins downstream of activated receptors, including mitogen-activated protein kinases, constituents of the phosphatidylinositol 3-kinase pathway, small GTPases, and β-arrestins. Reciprocally, some receptors influence IQGAP1 expression, subcellular localization, binding properties, and post-translational modifications. Importantly, the receptor:IQGAP1 crosstalk has pathological implications ranging from diabetes and macular degeneration to carcinogenesis. Here, we describe the interactions of IQGAP1 with receptors, summarize how they modulate signaling, and discuss their contribution to pathology. We also address the emerging functions in receptor signaling of IQGAP2 and IQGAP3, the other human IQGAP proteins. Overall, this review emphasizes the fundamental roles of IQGAPs in coupling activated receptors to cellular homeostasis.

## Teaser

David Sacks and colleagues review the crosstalk between cell surface receptors and the IQGAP scaffold proteins.

## Introduction

IQGAPs are evolutionarily conserved scaffold proteins with a multidomain architecture, allowing interactions with numerous, diverse proteins. Like most vertebrates, humans have three IQGAP proteins: IQGAP1, IQGAP2, and IQGAP3 (Briggs and Sacks, 2003; Box 1). IQGAP1 is the best characterized, with >150 interactors identified (Hedman et al., 2015). IQGAP1 scaffolds many signaling molecules in different signaling pathways (Table 1), including the mitogen-activated protein kinase (MAPK; Box 2) and the class I phosphatidylinositol 3-kinase (PI3K)/Akt (Box 3) networks. IQGAP1 also modulates small GTPase signaling by stabilizing their active or inactive forms, and/or by recruiting GTPase regulators (Table 1; Peng et al., 2021). By modulating intracellular signaling, IQGAP1 coordinates essential cellular processes like cell proliferation and migration, cytoskeletal dynamics, and vesicle trafficking (Smith et al., 2015). The cellular functions of IQGAP1 have physiological implications ranging from renal homeostasis and angiogenesis to insulin secretion (Hedman et al., 2015).

**Table 1.** Signaling molecules scaffolded by IQGAP1

Interactor	Binding site on IQGAP1	Effect of IQGAP1 binding	Reference
**AMPK**			
CaMKK2	IQ	Promotes AMPK activation	Hedman et al., 2021
AMPK	IQ		
**Hippo**			
MST2	IQ	Impairs MST2 and LATS1 kinase activity	Quinn et al., 2021
LATS1	IQ		
YAP	IQ	Inhibits YAP cotranscriptional activity	Sayedyahossein et al., 2016
**Insulin**			
IR	IQ	Stimulates insulin signaling	Chawla et al., 2017
IRS-1	RGCT and distal C-terminus		
**JAK-STAT**			
STAT1	n.d.	Activates the transcription factors STAT1/3	Chen et al., 2022
STAT3	n.d.		
**MAPK**			
B-Raf	IQ	Facilitates the MAPK phosphorylation cascade, ultimately activating ERK1/2	Ren et al., 2007
C-Raf	IQ and RGCT		Sbroggiò et al., 2011
MEK1	IQ		Roy et al., 2005
MEK2			
ERK1	WW		
ERK2			Roy et al., 2004
**PI3K/Akt**			
PI4KIIIα	n.d.	Facilitates the formation of PIP_3_	Choi et al., 2016; Tekletsadik et al., 2012
PIPKIα	IQ		
PI3K	WW and IQ		
mTorC1	N-terminal	Promotes Akt activation	
PDK1	n.d.		
Akt	IQ		
**Wnt**			
APC	RGCT	Scaffolds the degradation (APC) and activation (Dvl, PP2A) complexes of β-catenin	Watanabe et al., 2004
Dvl	aa 901–1060		Goto et al., 2013
PP2A	n.d.		Nakajima et al., 2005
β-catenin	RGCT		Kuroda et al., 1998
**Small GTPases**			
Arf1	IQ	Stimulates ERK activation	Hu et al., 2021
Arf6	n.d.	Stimulates Arf6-induced Rac1 activation	Hu et al., 2009
Cdc42	GRD	Stabilizes GTP-bound Cdc42	Kuroda et al., 1996
Rab27a	GRD	Regulates endocytosis	Kimura et al., 2013
Rac1	GRD	Stabilizes GTP-bound Rac1	Kuroda et al., 1996
Rac2	n.d.	Unknown	Meng et al., 2007
Ran1	n.d.	Regulates β-catenin transcriptional function	Goto et al., 2013
Rap1	IQ	Reduces Rap1 activation	Jeong et al., 2007
RhoA	n.d.	Modulates RhoA activation	Casteel et al., 2012
RhoC	C-terminal	Stimulates RhoC activation	Wu et al., 2011
RhoQ	n.d.	Unknown	Neudauer et al., 1998

Abbreviations: aa, amino acids; n.d., not determined.

Box 1 The three IQGAP proteins

The three human IQGAPs (IQGAP1, IQGAP2, and IQGAP3) share a similar multidomain composition, each containing a calponin homology domain (CHD), WW domain, IQ domain containing several IQ motifs, GTPase-activating protein (GAP)-related domain (GRD), and a RasGAP_C-terminal domain. IQGAPs are predominantly found in the cytosol, but also function at the plasma membrane to regulate receptor signaling and cell adhesion, at trafficking vesicles to coordinate endocytosis and exocytosis, and in the nucleus to influence transcription (Smith et al., 2015). All three proteins have common binding partners, including actin, the Ca^2+^-binding protein calmodulin, and the Rho GTPases Rac1 and Cdc42 (Hedman et al., 2015). However, their expression and functions differ. IQGAP1 is ubiquitously expressed, whereas IQGAP2 and IQGAP3 are predominantly expressed in the liver and brain, respectively (Wang et al., 2007). IQGAP1 and IQGAP3 are overexpressed in a wide array of neoplasms and are considered to be oncogenes (Wei and Lambert, 2021; White et al., 2009). In contrast, IQGAP2 expression is decreased in several malignancies, suggesting that it is a tumor suppressor (Wei and Lambert, 2021). The mechanisms that underly the opposite effects of IQGAP2 to IQGAP1 and IQGAP3 are unknown.

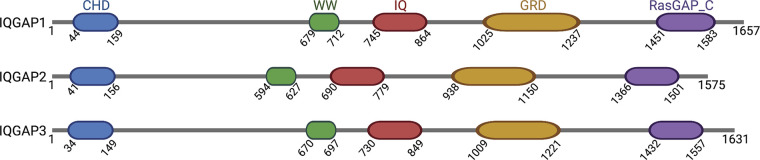

Box 2 IQGAP1 scaffolds the MAPK pathway

The MAPK pathway transduces extracellular signals to cellular responses, such as differentiation, proliferation, and apoptosis. The pathway is initiated by the GTPase Ras that, upon activation by receptors, activates Raf kinase. Then, Raf activates by phosphorylation MEK, which in turn phosphorylates ERK. Activated ERKs translocate to the nucleus, where they regulate expression of target genes to mediate cellular outcomes (Braicu et al., 2019). IQGAP1 facilitates the MAPK cascade by directly binding B-Raf, C-Raf, MEK1/2, and ERK1/2 (Ren et al., 2007; Roy et al., 2004; Roy et al., 2005; Sbroggiò et al., 2011). There are conflicting data regarding binding of Ras to IQGAP1, with the latest report indicating no interaction (Morgan et al., 2019). IQGAP1-mediated activation of ERK stimulates cell proliferation, migration, and invasion, and contributes to tumorigenesis (Jameson et al., 2013; White et al., 2011).

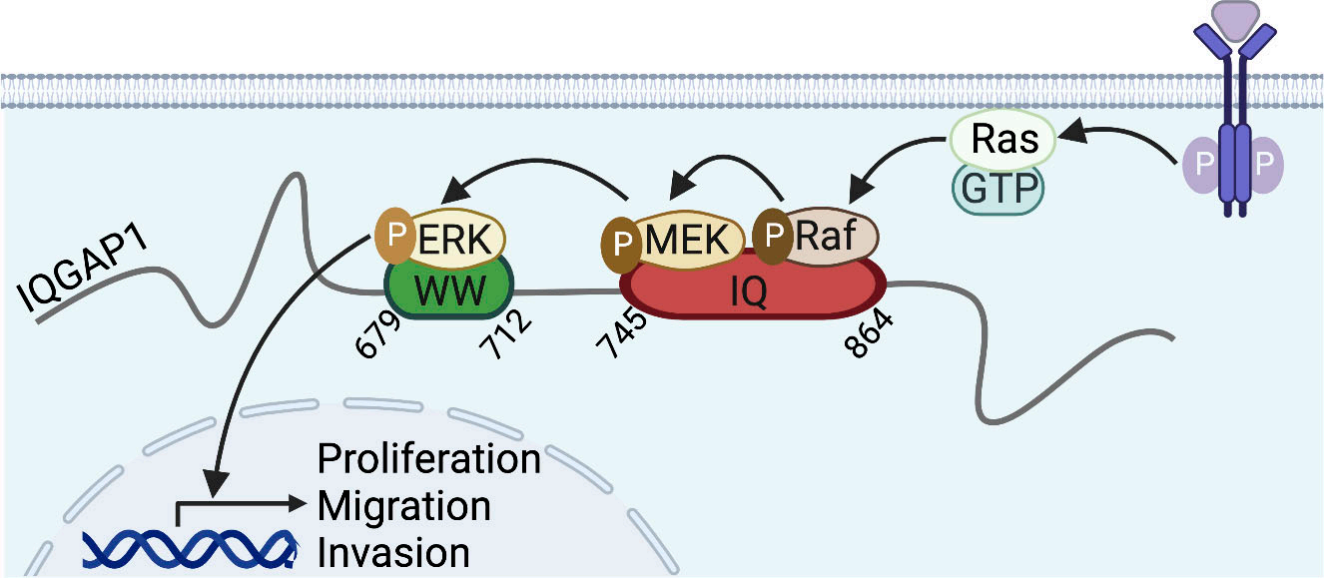

Box 3 IQGAP1 facilitates PI3K/Akt signaling

The PI3K/Akt pathway is important in cell cycle, growth, and proliferation. Receptor activation stimulates the pathway by recruiting and activating PI3K. Active PI3K catalyzes phosphorylation of the plasma membrane phosphatidylinositol-4,5-bisphosphate (PIP_2_) to phosphatidylinositol-3,4,5-trisphosphate (PIP_3_). PIP_3_ binds the kinases PDK1 and Akt, allowing PDK1-mediated activation of Akt that catalyzes phosphorylation of various targets to promote cellular responses (Jiang et al., 2020). IQGAP1 interacts with phosphatidylinositol 4-kinase III-α (PI4K) and type-I phosphatidylinositol phosphate kinase (PIPKI) that catalyze the formation of PIP_2_ from phosphatidylinositol (PI). Moreover, IQGAP1 binds PI3K, PDK1, and Akt to facilitate Akt activation (Choi et al., 2016). The potential for IQGAP1 to bind lipids may facilitate its recruitment at the plasma membrane to scaffold the pathway (Choi et al., 2013; Wang et al., 2019). IQGAP1 also associates with the mammalian target of rapamycin (mTOR), which promotes mTOR-catalyzed Akt activation (Chen et al., 2010). IQGAP1-mediated activation of Akt stimulates cell proliferation, migration, survival, and invasion, and drives carcinogenesis (Chen et al., 2010; Wei et al., 2020).

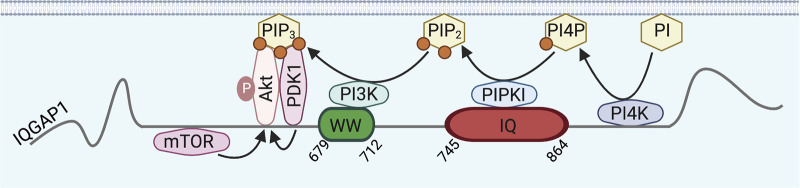

Cell surface receptors, particularly receptor tyrosine kinases (RTKs) and G-protein coupled receptors (GPCRs), are common IQGAP1 interactors (Table 2). IQGAP1 influences receptor signaling by (i) modulating receptor activation, expression and/or trafficking, and (ii) scaffolding signaling complexes at activated receptors. Reciprocally, some receptors influence IQGAP1 functions by modulating its subcellular localization, binding properties, and post-translational modifications (PTMs). Importantly, the receptor:IQGAP1 crosstalk affects cellular homeostasis and has pathological implications (Table 2). Here, we describe the interactions of diverse receptors with IQGAP1, address the implications at the molecular and cellular levels, and discuss their roles in disease development. The emerging participation of IQGAP2 and IQGAP3 in receptor signaling is also summarized.

**Table 2.** Cell surface receptors whose signaling is modulated by IQGAP, and implications in physiology and pathology

Receptor	Interaction in cells^a^	Direct binding^b^	Binding site on IQGAP1	Signaling outcome	Physiological and/or pathological implications	Reference^c^
**IQGAP1**						
**Receptor tyrosine kinases**						
Axl	Yes	Yes	IQ	Inhibits Axl activation and signaling to Akt	n.d.	Gorisse et al., 2018
EGFR	Yes	Yes	IQ	Promotes EGFR activation and signaling to ERK, Akt, STAT, and GTPases	Drives tumorigenesis	McNulty et al., 2011
FGFR1	Yes	Yes	n.d.	Stimulates N-WASP and B-Raf; promotes cell motility	n.d.	Benseñor et al., 2007
HER2	Yes	Yes	IQ	Sustains active HER2; induces resistance to trastuzumab	Promotes breast tumorigenesis	White et al., 2011
IGF-1R	n.d.	n.d.	IQ^d^	n.d.	n.d.	Salvi et al., 2022
IR	Yes	Yes	IQ	Increases IR signaling to Akt and ERK	Regulates glucose homeostasis; may contribute to diabetes	Chawla et al., 2017
MET	Yes	Yes	n.d.	Inhibits MET signaling; promotes Tyr phosphorylation of IQGAP1	Influences HGF/MET-induced tumorigenesis	Hedman et al., 2020
PDGFR-β	Yes	n.d.	n.d.	Modulates focal adhesion assembly and cell motility	Coordinates vascular repair	Kohno et al., 2013
VEGFR2	Yes	Yes	n.d.	Loosens cell adhesion and increases cell migration; stimulates angiogenesis and neurogenesis	Contributes to macular degeneration and cancer development	Yamaoka-Tojo et al., 2004
**G protein–coupled receptors**						
CXCR2	Yes	Yes	CHD	May inhibit chemotaxis	n.d.	Neel et al., 2011
CXCR4	Yes	n.d.	n.d.	Increases receptor trafficking, ERK activation, and cell migration	n.d.	Bamidele et al., 2015
DOR1	n.d.	n.d.	n.d.	Increases receptor trafficking and ERK activation	n.d.	Bamidele et al., 2015
DP1	Yes	n.d.	n.d.	Regulates DP1 trafficking; increases ERK activation	n.d.	Fréchette et al., 2021
ET-1R	n.d.	n.d.	n.d.	Modulates GTPase activation; enhances cell migration, invasion, and metastasis	May promote ovarian carcinogenesis	Chellini et al., 2019
GPR161	Yes	n.d.	n.d.	Enhances cell migration and proliferation	May contribute to breast cancer	Feigin et al., 2014
KISS1R	Yes	n.d.	n.d.	Stimulates EGFR transactivation	n.d.	Cvetkovic et al., 2013
LGR4	Yes	n.d.	GRD	Increases canonical and non-canonical Wnt signaling	n.d.	Carmon et al., 2014
LGR5	Yes	n.d.	GRD and C-terminus	Reduces IQGAP1 phosphorylation; increases Rac1 and actin binding	n.d.	Carmon et al., 2017
LPA1	Yes	n.d.	n.d.	Increases cell migration and invasion	n.d.	Alemayehu et al., 2013
M3-mAChR	n.d.	n.d.	n.d.	Increases active Rac1; may couple M3-mAChR to actin cytoskeleton	n.d.	Ruiz-Velasco et al., 2002
MOR1	n.d.	n.d.	n.d.	Stimulates Rac1 and ERK activation	n.d.	Civciristov et al., 2019
**Receptor serine/threonine kinases**						
TGFβR2	Yes	Yes	aa 1503–1657	Promotes TGFβR2 degradation; inhibits TGFβR2 signaling	May constrain liver and bladder tumor growth	Liu et al., 2013
**Glutamate-gated ion channels**						
AMPAR (GluR4)	Yes	n.d.	N-terminal	May regulate cell surface targeting of GluR4	May participate in cognitive physiology and pathology	Nuriya et al., 2005
NMDAR (NR2A, NR2B)	Yes	n.d.	n.d.	Promotes cell surface targeting of NR2A; stimulates ERK signaling		Gao et al., 2011
**Adhesion receptors**						
β1-integrin	Yes	n.d.	n.d.	Couples β1-integrin to Rac1, RhoA, and Arf6 GTPases	n.d.	Nakajima et al., 2005
β3-integrin	Yes	Yes	n.d.	Promotes cortical actin arrangements	Controls vascular barrier protection	Bhattacharya et al., 2012
CD44	Yes	n.d.	n.d.	Links CD44 to the cytoskeleton and ERK signaling	Increases ovarian tumor cell migration	Bourguignon et al., 2005
E-cadherin	Yes	Yes	RGCT	Dissociates adherens junctions	n.d.	Kuroda et al., 1998
N-cadherin	Yes	n.d.	n.d	Stimulates ERK1/2 signaling; regulates cell-cell adhesion	Participates in fear memory and spermatogenesis	Lui et al., 2005
VE-cadherin	Yes	n.d.	n.d.	Destabilizes adherens junctions	n.d.	Yamaoka-Tojo et al., 2006
**T cell receptors**						
OX40	Yes	n.d.	C-terminal	Restrains OX40 cosignaling	May coordinate inflammation	Okuyama et al., 2020
TCR	n.d.	n.d.	n.d.	Negatively regulates TCR-mediated signaling		Gorman et al., 2012
**Receptor protein tyrosine phosphatases**						
PTPμ	Yes	Yes	n.d.	Regulates GTPase-dependent functions of IQGAP1 and neurite outgrowth	n.d.	Phillips-Mason et al., 2006
**IQGAP2**						
DP1	n.d.	n.d.	n.d.	Inhibits ERK	n.d.	Fréchette et al., 2021
IFN-α receptor	n.d.	n.d.	n.d.	Activates NF-κB-mediated gene expression	May have antiviral actions	Brisac et al., 2016
LGR4	Yes	n.d.	n.d.	n.d.	n.d.	Carmon et al., 2014
PAR	n.d.	n.d.	n.d.	Modulates cytoskeletal dynamics	n.d.	Schmidt et al., 2003
VEGFR2	n.d.	n.d.	n.d.	Stimulates VEGF production, which activates VEGFR2 signaling to Akt	Increases angiogenesis in breast cancer	Kumar et al., 2022
**IQGAP3**						
DP1	n.d.	n.d.	n.d.	Activates ERK	n.d.	Fréchette et al., 2021
EGFR	n.d.	n.d.	n.d.	Stimulates EGFR activation and signaling to ERK	n.d.	Yang et al., 2014
FGFR1	n.d.	n.d.	n.d.	Activates ERK	May coordinate embryonic development	Fang et al., 2015
LGR4	Yes	n.d.	n.d.	Enhances RSPO/LGR4 signaling to Wnt/β-catenin	n.d.	Carmon et al., 2014
NGFR	n.d.	n.d.	n.d.	Triggers formation of cell extensions	n.d.	Caro-Gonzalez et al., 2012

Abbreviations: aa, amino acids; n.d., not determined.

a Interaction in cells was demonstrated by co-immunoprecipitation or pull-down from cell lysates, or by colocalization or proximity ligation assay in intact cells.

b Direct binding was demonstrated using pure proteins.

c Only the initial publication is cited here.

d Interaction was suggested from in silico molecular docking analysis only.

## IQGAP1 integrates receptor tyrosine kinase signaling

RTKs are characterized by intracellular tyrosine kinase activity. Activation upon binding of their cognate ligand most commonly induces RTK dimerization and autophosphorylation. Proteins are then recruited to the receptor primarily through Src homology 2 (SH2) and phosphotyrosine-binding (PTB) domains to initiate signaling (Hubbard and Miller, 2007). RTKs control essential cellular processes including proliferation, migration, and differentiation (Pawson, 2002). IQGAP1 has been documented to influence signaling of 9 of the 58 human RTKs (Table 2). Usually, IQGAP1 stimulates RTK signaling (EGFR, FGFR1, HER2, IGF-1R, IR, PDGFR-β, VEGFR2), but inhibits Axl and MET. IQGAP1 couples RTK activation to intracellular signaling by scaffolding the MAPK (Box 2) and PI3K/Akt (Box 3) pathways, and by recruiting small GTPases.

### IQGAP1 promotes tumorigenic signaling of EGFR and HER2

The erythroblastic leukemia viral oncogene homolog (ErbB) sub-family of RTKs comprises four structurally related members that mediate cell proliferation, differentiation, and migration (Appert-Collin et al., 2015). IQGAP1 has been reported to bind two ErbB members, epidermal growth factor receptor (EGFR; McNulty et al., 2011) and human epidermal growth factor receptor 2 (HER2; White et al., 2011), which enhances their signaling and promotes tumorigenesis.

**EGFR: **The EGFR:IQGAP1 complex was identified by mass spectrometry (Blagoev et al., 2003), and later confirmed in A431 epidermoid carcinoma (McNulty et al., 2011), breast carcinoma (Chen et al., 2019), and ovarian cancer cells (Chen et al., 2022). Binding occurs directly, at the IQ domain of IQGAP1 (McNulty et al., 2011), but also via the adaptor protein ShcA (Smith et al., 2010). EGF does not modulate the EGFR:IQGAP1 interaction; IQGAP1 binds both quiescent and activated receptors (McNulty et al., 2011).

**Figure 1.**
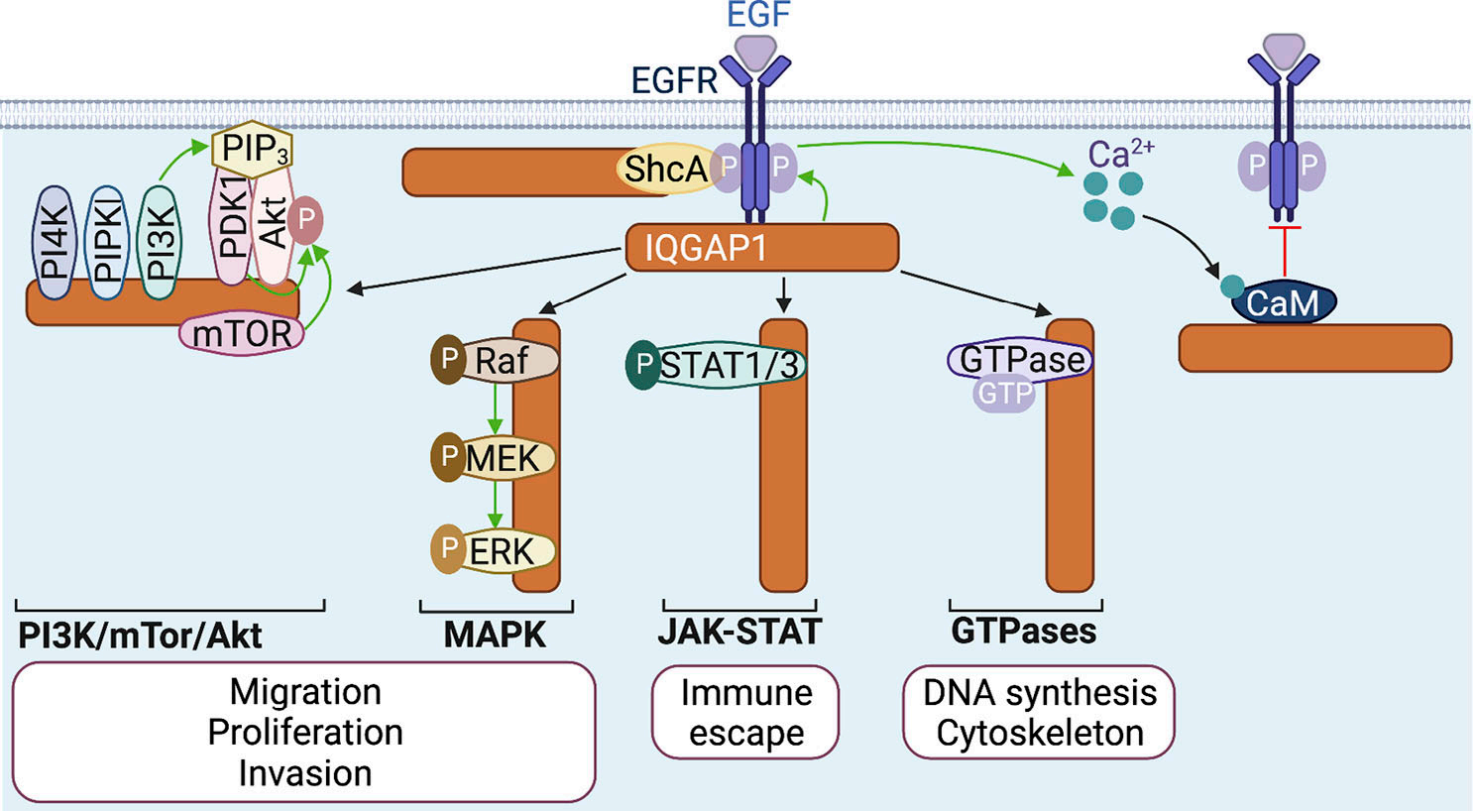
**IQGAP1 scaffolds signaling pathways upon activation of EGFR.** IQGAP1 binds to EGFR, both directly and via the adaptor protein ShcA. EGF stimulates IQGAP1-mediated scaffolding of the PI3K/mTor/Akt and MAPK pathways, which activates Akt and ERK, respectively. IQGAP1 also facilitates EGF-stimulated activation of the transcription factors STAT1 and STAT3, and stabilizes the GTP-bound forms of the GTPases Cdc42, RhoA, and RhoC. The functional implications of IQGAP1-mediated signaling are indicated below each pathway. Ca^2+^/calmodulin (CaM), generated by EGF/EGFR-induced increase of cytosolic Ca^2+^, binds IQGAP1 and prevents its binding to EGFR, providing negative feedback. Green and red arrows represent stimulation and inhibition, respectively. The figure was generated in BioRender.

IQGAP1 binding stimulates EGFR signaling and couples EGFR activation to the MAPK pathway (Fig. 1). IQGAP1 knockdown reduces EGF-induced autophosphorylation of EGFR (McNulty et al., 2011; Monteleon et al., 2015). Moreover, IQGAP1 depletion decreases EGF-stimulated activation of the MAPK proteins B-Raf, MEK1/2, and ERK1/2 (Box 2; Ren et al., 2007; Roy et al., 2004; Roy et al., 2005). Interestingly, overexpression of IQGAP1 also impairs EGF-stimulated MAPK activation (Roy et al., 2004; Roy et al., 2005), probably by increasing formation of non-functional complexes comprising only one MAPK protein. EGF stimulates and inhibits IQGAP1 binding to MEK1 and MEK2, respectively (Roy et al., 2005), suggesting that IQGAP1 activates ERK preferentially via MEK1.

IQGAP1 also links EGFR to PI3K/Akt signaling (Fig. 1). EGF induces scaffolding by IQGAP1 of PI4KIIIα, PIPKIα, and PI3K, increasing PIP_3_ production. Moreover, EGF increases PDK1 and Akt binding to IQGAP1, thereby promoting PDK1-catalyzed activation of Akt (Box 3; Choi et al., 2016). IQGAP1 also binds mTORC1, augmenting mTOR-catalyzed Akt activation by EGF (Tekletsadik et al., 2012). IQGAP1 depletion from cells and mice decreases EGF-stimulated Akt activation (Choi et al., 2016; Wei et al., 2020). Coupling of EGF/EGFR to Akt by IQGAP1 has implications in cancer. Loss of IQGAP1 from mice with EGFR-driven head and neck cancer reduces oncogenic Akt upregulation, correlating with improved prognosis (Wei et al., 2020). Moreover, deletion of the third of four IQ motifs (IQ3) from IQGAP1 abrogates binding to PI3K/Akt, which reduces EGF-stimulated migration and invasion of squamous carcinoma cells. Specific scaffolding of the PI3K/Akt, but not the MAPK, pathway at the IQ3 motif of IQGAP1 provides opportunities for therapies targeted at carcinogenic PI3K/Akt signaling (Chen et al., 2019).

IQGAP1 also couples EGF to GTPase-mediated signaling. EGF increases binding of the small GTPases Cdc42, RhoA, and RhoC to IQGAP1, which stabilizes their GTP-bound, active forms. Active Cdc42 promotes actin polymerization while active RhoA and RhoC stimulate DNA synthesis in breast cancer cells, with possible carcinogenic implications (Fig. 1; Casteel et al., 2012; Erickson et al., 1997). Additionally, IQGAP1 stimulates EGF-induced activation of the JAK-STAT pathway, a signaling module where JAK non-receptor tyrosine kinases activate STAT transcription factors to promote expression of critical mediators of cancer and inflammation (Hu et al., 2021). EGF stimulates the interaction of IQGAP1 with STAT1 and STAT3 (Chen et al., 2022). This increases nuclear activity of STAT1/3, which upregulates expression of PD-L1, the ligand of the T-cell receptor PD-L. Because PD-L/PD-L1 signaling inactivates T-cells, this mechanism favors immune escape of tumor cells (Fig. 1; Chen et al., 2022). Interestingly, IQGAP1 also promotes EGF-induced nuclear translocation of β-catenin, a component of the cadherin complex that is a signaling transducer in the Wnt pathway (Osman et al., 2020), implying that IQGAP1 may also couple EGFR to expression of Wnt target genes.

EGFR:IQGAP1 crosstalk influences cell division. In mitotic epithelial cells, EGFR localizes at basolateral membranes where it recruits IQGAP1. Once there, IQGAP1 controls orientation of the mitotic spindle to ensure proper cell division. Disrupting basolateral localization of IQGAP1 or EGFR causes misorientation of the mitotic spindle and alters the formation of single-lumen cysts, impacting tissue morphology (Bañón-Rodríguez et al., 2014). Interestingly, EGFR inhibitors used to treat EGFR-activated lung cancer upregulate IQGAP1 expression, which correlates with increased vascular permeability. These findings suggest vascular functions for EGFR:IQGAP1, which could contribute to purpuric drug eruptions, an adverse event commonly observed during EGFR inhibitor therapies (Sheen et al., 2020).

EGFR:IQGAP1 complexes are negatively regulated by Ca^2+^. Active EGFR increases cytosolic Ca^2+^ concentration by inducing Ca^2+^ release from the endoplasmic reticulum, which augments the action of the Ca^2+^-binding protein calmodulin bound to Ca^2+^ (Bryant et al., 2004). In turn, Ca^2+^/calmodulin, which binds to the IQ domain of IQGAP1 (Li and Sacks, 2003), abrogates formation of EGFR:IQGAP1 complexes, thereby altering EGF-stimulated, IQGAP1-mediated signaling (Fig. 1; McNulty et al., 2011). IQGAP1 also coordinates ERK1/2-catalyzed phosphorylation of EGFR, which could provide an additional negative regulatory loop (Casar et al., 2009).

**HER2: **Unlike EGFR, HER2 has no ligand binding domain; it is activated by heterodimerization with other ligand-activated ErbB members. HER2 overexpression causes HER2-positive breast cancer (Yarden, 2001). IQGAP1 binds HER2 in HER2-positive breast cancer cells. IQGAP1 knockdown alters HER2 stability, activation, and signaling to Akt, which reduces breast cancer cell proliferation (White et al., 2011). This indicates that the HER2:IQGAP1 complex promotes breast tumorigenesis. Importantly, IQGAP1 overexpression in HER2-positive breast and gastric cancer cells induces resistance to trastuzumab, a therapeutic monoclonal antibody targeted at HER2, likely by stabilizing active HER2 (Arienti et al., 2016; White et al., 2011). IQGAP1 is also recruited to activated HER2 via the adaptor protein ShcA, which has been proposed to couple HER2 to cytoskeletal rearrangements (Smith et al., 2010).

### IQGAP1 stimulates VEGF, PDGF, and FGF signaling to enhance cell motility

IQGAP1 regulates cell motility, in part by binding actin and the GTPases Cdc42 and Rac1 (Mataraza et al., 2003; Noritake et al., 2005). IQGAP1 also coordinates cell motility by coupling vascular endothelial growth factor (VEGF), platelet-derived growth factor (PDGF), and fibroblast growth factor (FGF) stimulation to cytoskeletal rearrangements.

**Figure 2.**
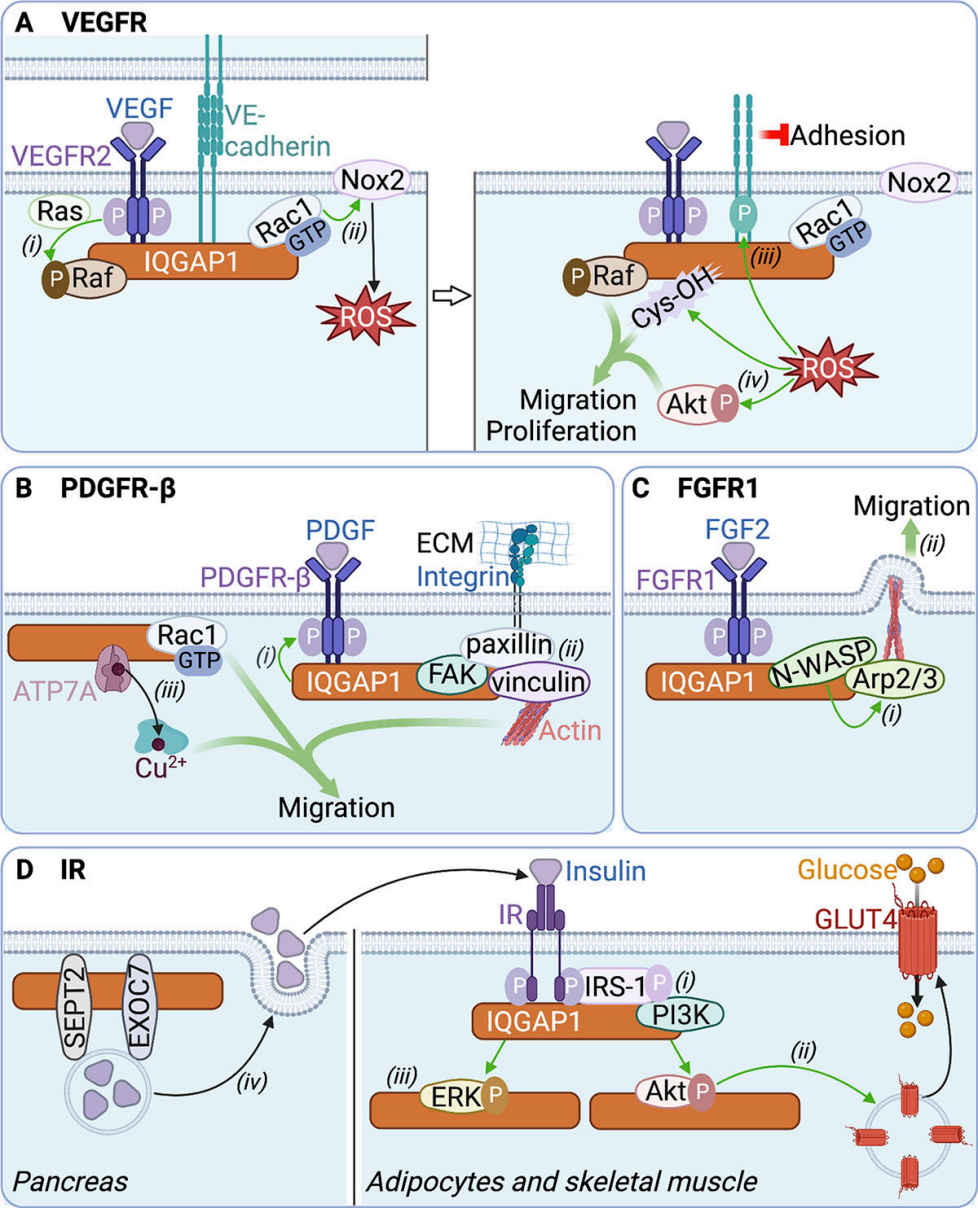
**IQGAP1 scaffolds signaling complexes at activated receptor tyrosine kinases. (A)** VEGFR2: VEGFR2 activated by VEGF binds IQGAP1. IQGAP1 couples VEGF/VEGFR2 to (i) Raf activation and (ii) activation of the ROS-producing Nox2 oxidase by Rac1-GTP. (iii) ROS stimulate phosphorylation of VE-cadherin, which loosens cell adhesion. (iv) ROS also activate Akt and induce cysteine oxidation (Cys-OH) of IQGAP1. Cys-OH, active Akt, and active Raf drive cell migration and proliferation. **(B)** PDGFR-β: PDGFR-β activated by PDGF binds IQGAP1. (i) IQGAP1 stimulates PDGFR-β activation. (ii) IQGAP1 assembles at activated PDGFR-β a complex containing paxillin, vinculin, and focal adhesion kinase (FAK) that form integrin-mediated focal adhesion structures. (iii) IQGAP1 also recruits Rac1-GTP and the copper transporter ATP7A (during trafficking to the plasma membrane), which delivers Cu^2+^ to Cu^2+^-dependent enzymes. These events drive cell migration. ECM, extracellular matrix. **(C)** FGFR1: FGFR1 activated by FGF2 binds IQGAP1. (i) IQGAP1 recruits N-WASP and Arp2/3, which facilitates Arp2/3 activation by N-WASP (ii) to promote lamellipodium formation and cell migration. **(D)** Insulin receptor: IQGAP1 binds to IR and IRS-1, which (i) initiates the phosphorylation-dependent recruitment of PI3K to IRS-1 and facilitates Akt activation. (ii) Active Akt induces translocation of GLUT4 to the plasma membrane, enabling glucose entry into adipocytes and skeletal muscle cells. (iii) IQGAP1 also facilitates ERK activation by insulin. (iv) In the pancreas, IQGAP1 binding to the exocyst protein EXOC7 and septin SEPT2 promotes exocytic release of insulin. Green and red arrows represent stimulation and inhibition, respectively. The figure was generated in BioRender.

**VEGFR2: **VEGF receptor-2 (VEGFR2), predominantly expressed in vascular endothelial (VE) cells, stimulates cell proliferation and migration to promote angiogenesis. VEGF induces binding of IQGAP1 to VEGFR2, in complex with Rac1. IQGAP1 stabilizes GTP-bound Rac1, which activates the NAD(P)H oxidase Nox2, leading to the generation of reactive oxygen species (ROS; Fig. 2 A; Ikeda et al., 2005; Yamaoka-Tojo et al., 2004). ROS stimulate tyrosine phosphorylation of VE-cadherin, which is also found in the VEGFR2:IQGAP1 complex, thereby loosening cell–cell adhesion (Yamaoka-Tojo et al., 2006). ROS produced by Nox2 also activate Akt to promote cell proliferation and migration (Yamaoka-Tojo et al., 2004). Moreover, ROS induce oxidation of IQGAP1 cysteine residues into cysteine sulfenic acid, a redox intermediate suggested to enhance cell migration (Kaplan et al., 2011). IQGAP1 also promotes VEGF-stimulated activation of B-Raf, which increases cell proliferation (Fig. 2 A; Meyer et al., 2008). Participation in VE cell adhesion, migration, and proliferation suggests that VEGFR2:IQGAP1 coordinates angiogenesis. Consistently, IQGAP1 is required for VEGF-stimulated tube formation from 3D-cultured VE cells (Yamaoka-Tojo et al., 2006). In vivo, vascular injury in rats upregulates IQGAP1 and VEGFR2 expression (Yamaoka-Tojo et al., 2004), while IQGAP1 knockdown reduces VEGF-stimulated angiogenesis of the highly vascularized extraembryonic chorioallantoic membrane of fertilized chicken eggs (Meyer et al., 2008). In neural stem cells, VEGF promotes binding of IQGAP1 to active Cdc42 and Rac1, and to the microtubule-associated protein Lis1. These associations augment migration and differentiation of neural progenitor cells, thereby contributing to neurogenesis (Balenci et al., 2007).

The functions of VEGFR2:IQGAP1 in cell motility may have pathological consequences. In the eye, activation of Rac1 by the VEGFR2:IQGAP1 complex promotes migration of choroidal endothelial cells. The migrating cells produce choroidal neovascularization, ultimately causing macular degeneration (Wang et al., 2020). Consistent with this observation, both active Rac1 and choroidal neovascularization are reduced in IQGAP1-null mice. In esophageal tumors, IQGAP1 overexpression enhances VEGF expression and VEGFR2 activation, which stimulates angiogenesis to favor tumor progression. Interestingly, Akt and ERK inhibitors abrogate the effects of IQGAP1 overexpression. Together, this implies that IQGAP1 overexpression in esophageal cancer promotes Akt/ERK-mediated oncogenic VEGF/VEGFR2 signaling (Li et al., 2018). Similarly, IQGAP1 overexpression in gastric carcinoma cells stimulates VEGF expression and secretion, which may drive carcinogenic epithelial-to-mesenchymal transition (EMT; Liu et al., 2019). Finally, IQGAP1 promotes VEGF-stimulated proliferation of myeloma cells (Ma et al., 2013), further implicating IQGAP1-mediated VEGF/VEGFR2 signaling in tumorigenesis.

Analogous to VEGF, angiopoietin-1, a ligand of the endothelial RTK Tie2, stimulates Rac1 binding to IQGAP1 and stabilizes Rac1-GTP. IQGAP1 knockdown, by decreasing angiopoietin-1-stimulated activation of Rac1, reduces endothelial barrier functions (David et al., 2011). Whether these effects are modulated by Tie2:IQGAP1 binding remains to be addressed.

**PDGFR-β: **The PDGF receptor-β (PDGFR-β) is an essential regulator of hematopoiesis and angiogenesis during development (Chen et al., 2013). In vascular smooth muscle cells (VSMCs), PDGF promotes IQGAP1 binding to PDGFR-β, in complex with the focal adhesion proteins vinculin, paxillin, and focal adhesion kinase (Fig. 2 B). In this complex, IQGAP1 stimulates PDGFR-β activation. Further, IQGAP1 facilitates the formation of focal adhesion structures to enhance cell migration (Kohno et al., 2013). PDGF also induces IQGAP1 binding to the copper transporter ATP7A and Rac1 in lipid rafts and at the leading edge of VSMCs (Ashino et al., 2018). This complex increases cell motility by stabilizing GTP-bound Rac1 and activating Cu^2+^-dependent enzymes (Fig. 2 B; Ashino et al., 2018). IQGAP1 depletion decreases PDGF-stimulated B-Raf and Akt activation (Choi et al., 2016; Ren et al., 2007), suggesting that IQGAP1 bridges activated PDGFR-β to both the MAPK and PI3K/Akt pathways.

IQGAP1-mediated PDGF/PDGFR-β signaling, by driving VSMC migration, has been suggested to promote vascular repair. IQGAP1 expression increases during repair of vascular injury in mice, whereas repair is defective in IQGAP1-null mice (Kohno et al., 2013). PDGFR-β:IQGAP1 similarly enhances migration of both VSMCs and VE cells during sepsis, revealing functions in the repair of sepsis-associated vascular damage (Zheng et al., 2019a; Zheng et al., 2019b). Interestingly, PDGF increases IQGAP1 expression by downregulating IQGAP1-targeted microRNAs (miR-23b and miR-125a-5p), causing oncogenic overexpression of IQGAP1 in lung cancer (Naidu et al., 2017).

**FGFR1: **The FGF receptor-1 (FGFR1) controls embryo development and adult tissue homeostasis by regulating cell proliferation and differentiation (Groth and Lardelli, 2002). FGF2 induces binding of IQGAP1 to FGFR1 in bovine kidney cells. Both neuronal Wiskott-Aldrich syndrome protein (N-WASP) and actin-related protein 2/3 (Arp2/3) are in the FGFR1:IQGAP1 complex, which facilitates activation of Arp2/3 by N-WASP (Fig. 2 C; Benseñor et al., 2007). Activated Arp2/3 induces actin polymerization and branching, which stimulates the formation of lamellipodia and increases cell motility. Depletion of IQGAP1 impairs the ability of FGF2 to activate B-Raf (Ren et al., 2007), implying that IQGAP1 couples FGFR1 to MAPK activation. The physiological and potential pathological implications of FGFR1:IQGAP1-stimulated cell motility are unknown.

### IQGAP1 stimulates insulin signaling

Increases in blood glucose concentrations result in secretion of insulin and activation of the insulin receptor (IR). Activated IR recruits and phosphorylates insulin receptor substrate-1 (IRS-1), which recruits signaling proteins, including PI3K, which activates Akt to control GLUT4-mediated glucose uptake (Saltiel, 2021). The IQGAP1 IQ region and C-terminal tail directly bind IR and IRS-1, respectively (Chawla et al., 2017; Fig. 2 D). In cultured adipocytes, insulin enhances the interaction of IQGAP1 with IRS-1 and PI3K (Hansson et al., 2019). Loss of IQGAP1 impairs the recruitment of PI3K to IRS-1, which decreases Akt activation. IQGAP1 depletion also reduces IR coupling to ERK activation (Fig. 2 D; Chawla et al., 2017). In silico molecular docking analysis suggests that IQGAP1 also binds to the insulin-like growth factor-1 receptor (IGF-1R; Salvi et al., 2022). IQGAP1 knockdown reduces IGF-1-induced stimulation of ERK and Akt (Choi et al., 2016; Roy et al., 2004). These findings indicate that IQGAP1 couples IR and IGF-1R activation to the PI3K/Akt and MAPK cascades. Furthermore, IQGAP1 stimulates insulin secretion from pancreatic β-cells by interacting with the exocyst protein EXOC7 and septin-2 (Fig. 2 D). This mechanism is regulated by active Cdc42, which dissociates the IQGAP1:EXOC7:SEPT2 complex to restrict insulin secretion (Rittmeyer et al., 2008). Importantly, IQGAP1-null mice display impaired insulin-stimulated PIP_3_ synthesis and Akt activation, as well as impaired glucose homeostasis and insulin resistance (Chawla et al., 2017; Choi et al., 2016). Moreover, the abundance of IQGAP1 in adipocytes from type 2 diabetes patients is lower than in non-diabetic subjects (Jufvas et al., 2016). Collectively, these data implicate IQGAP1 in insulin-mediated glucose regulation, suggesting it may be an appealing target for diabetes therapy.

### IQGAP1 inhibits signaling of the RTK Axl

In contrast to the other RTKs, IQGAP1 impairs Axl signaling. Axl is activated by Gas6 to control cell survival, proliferation, migration, and invasion (Colavito, 2020). IQGAP1 directly binds to Axl in breast cancer cells. Unlike the other RTK:IQGAP1 interactions, which are increased or not influenced by ligand stimulation, Gas6 inhibits Axl:IQGAP1 association (Gorisse et al., 2018). Moreover, IQGAP1 reduces Axl activation, both by decreasing Gas6-mediated autophosphorylation and by altering heterodimerization with EGFR, which transactivates Axl independently of Gas6. IQGAP1 also impairs Gas6/Axl coupling to Akt and reduces Gas6/Axl-induced expression of matrix metalloproteases. In contrast, IQGAP1 does not modulate Gas6/Axl signaling to ERK (Gorisse et al., 2018), implying that IQGAP1 couples Axl only to selective pathways. The physiological consequences of the inhibitory binding of IQGAP1 to Axl remain unexplored. Because both IQGAP1 and Axl participate in immune cell activities (Abel et al., 2015; Tanaka and Siemann, 2020) and carcinogenesis (Wei and Lambert, 2021; Wium et al., 2021), one might speculate that those processes could be modulated by Axl:IQGAP1.

### IQGAP1 integrates HGF/MET signaling

MET is activated by the hepatocyte growth factor (HGF) to control cell growth, proliferation, survival, and motility, which drives embryogenesis and wound healing, but also tumorigenesis (Desole et al., 2021). IQGAP1 binds directly to the MET receptor, and this is increased by HGF (Thines et al., 2023). IQGAP1 knockdown increases HGF-stimulated MET activation and coupling to Akt and ERK in hepatocellular carcinoma cells (Delgado et al., 2021; Thines et al., 2023), indicating that it inhibits MET signaling. In contrast, IQGAP1 promotes HGF-stimulated colon cancer cell invasion (Hayashi et al., 2010), implying that the functions of IQGAP1 in HGF/MET signaling may be cell-dependent. HGF also stimulates the interaction of IQGAP1 with several other proteins: (i) with the Rac1/Cdc42 guanine nucleotide exchange factor Asef, the actin binding protein cortactin, and the microtubule binding protein EB1 to enhance endothelial barrier function (Tian et al., 2015; Tian et al., 2014); (ii) with E-cadherin/β-catenin and the kinase PAK6 to loosen cell–cell adhesion (Fram et al., 2014; Shimao et al., 2002); and (iii) with the GTPase Arf6 to enhance glioma cell migration (Hu et al., 2009). Some of these interactions are suggested to result from the translocation of IQGAP1 to the plasma membrane induced by HGF (Hu et al., 2009; Shimao et al., 2002). Interestingly, depletion of IQGAP1 upregulates HGF expression (Liu et al., 2013), suggesting a feedback mechanism by IQGAP1 on HGF/MET signaling.

## IQGAP1 participates in G protein–coupled receptor signaling

GPCRs translate extracellular ligand binding to intracellular signals (Wootten et al., 2018). The human genome encodes ∼800 GPCRs, which modulate multiple physiological processes and are targeted by ∼35% of prescription drugs (Hauser et al., 2017). Ligand binding activates GPCRs by conformational shifts, which create an intracellular binding pocket that engages heterotrimeric G_αβγ_ proteins (Hilger et al., 2018). Activated GPCRs induce GTP binding to G_α_, which then dissociates from G_βγ_. G_α_ and G_βγ_ separately mediate signaling (Weis and Kobilka, 2018). Arrestin adaptor proteins can either activate or inhibit GPCR signaling independently from G proteins (Gurevich and Gurevich, 2019). GPCR signaling is also modulated by recruitment of other proteins, including scaffolds that assemble complexes at the receptor. IQGAP1 participates in signaling by 12 GPCRs, with implications in cell physiology and pathology (Table 2).

### CXC chemokine receptors

CXC chemokine receptors (CXCRs) bind cytokines of the CXC family to mediate inflammatory and angiogenic functions (Vandercappellen et al., 2008). CXCR2, one of the six human CXCRs, binds directly to the CHD of IQGAP1 and colocalizes with IQGAP1 at the leading edge of polarized neutrophils (Neel et al., 2011). The interaction is reduced by the CXCR2 ligand interleukin-8 (IL-8). Interestingly, exogenous expression of the IQGAP1 CHD inhibits IL-8/CXCR2-mediated chemotaxis in HEK293 cells. While the IQGAP1 CHD did not block CXCR2 binding, the authors postulate that IQGAP1 may negatively regulate CXCR2-mediated chemotaxis (Neel et al., 2011).

**Figure 3.**
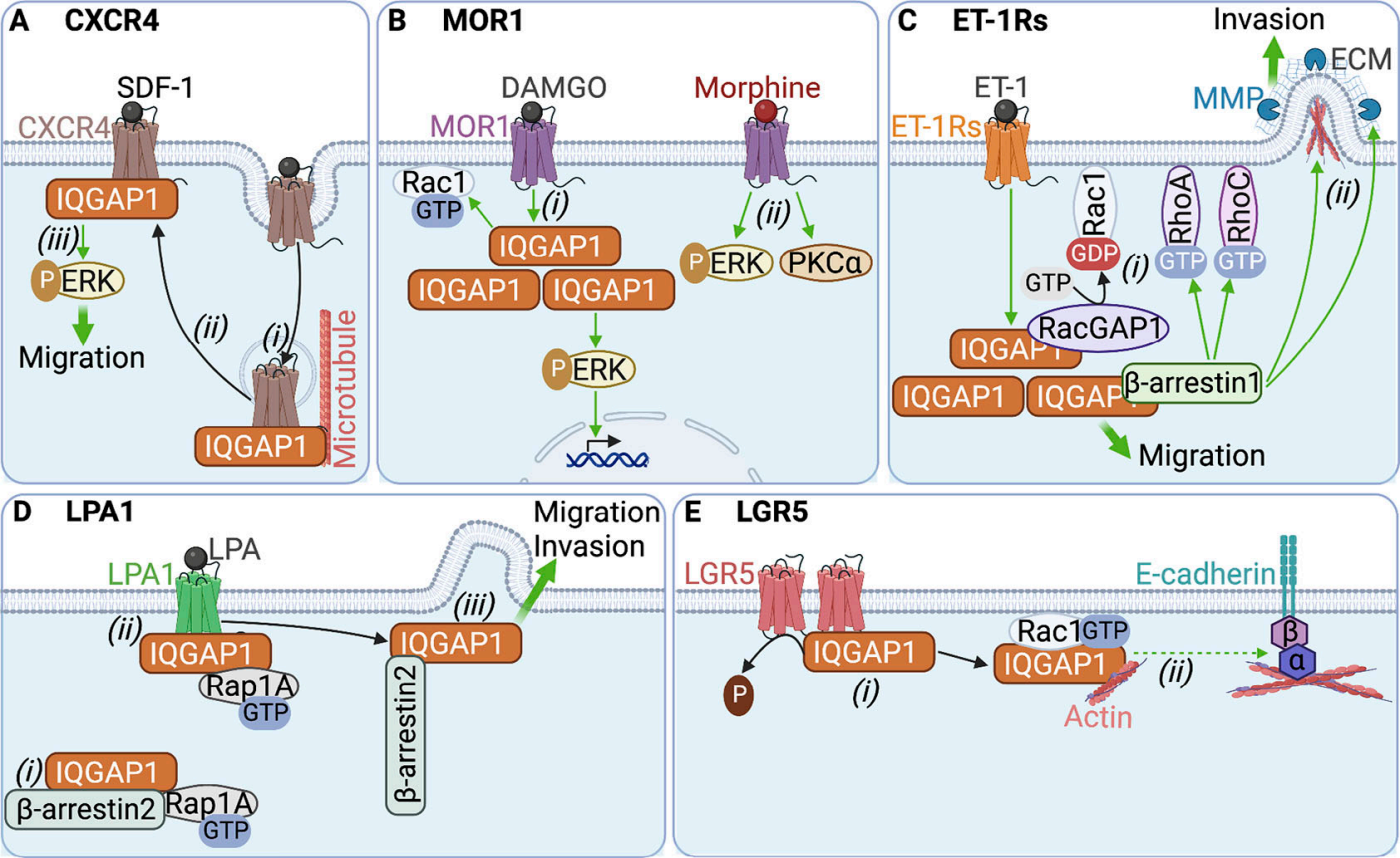
**IQGAP1 integrates GPCR signaling. (A)** CXCR4: (i) SDF-1 induces recruitment of IQGAP1 to CXCR4-containing endosomes. (ii) By binding α-tubulin in microtubules, IQGAP1 coordinates post-endocytic trafficking of CXCR4. (iii) At the cell surface, IQGAP1 stimulates SDF-1/CXCR4-induced ERK activation to promote cell migration. **(B)** MOR1: (i) MOR1 activation by DAMGO increases the amount of IQGAP1 and stimulates active Rac1 and nuclear ERK. (ii) Morphine activates ERK and PKCα independently of IQGAP1. **(C)** ET-1Rs: (i) Stimulation of ET-1Rs by ET-1 increases the amount of IQGAP1 and induces IQGAP1 binding to β-arrestin1, and RacGAP1. This reduces active Rac1 and increases active RhoA and RhoC, which stimulates cell migration. (ii) The IQGAP1:β-arrestin1 complex also promotes invadopodium formation and degradation of the extracellular matrix (ECM) by matrix metalloproteinases (MMPs), thereby coordinating cell invasion. **(D)** LPA1: (i) IQGAP1 binds constitutively to Rap1A and β-arrestin2. (ii) LPA enhances binding of active Rap1A and IQGAP1 to LPA1. (iii) LPA also induces colocalization of β-arrestin2 and IQGAP1 at the leading edge of migrating cells, which increases cell migration and invasion. **(E)** LGR5: (i) IQGAP1 binding to overexpressed LGR5 reduces IQGAP1 phosphorylation, which increases its association with Rac1-GTP and actin. (ii) Because active Rac1 decreases IQGAP1:β-catenin interaction, this mechanism is suggested to promote the formation of the E-cadherin:β-catenin:α-catenin complex and to increase actin cross-linking. Green arrows represent stimulation, while dashed arrows depict speculative mechanisms not confirmed by experimental data. The figure was generated in BioRender.

IQGAP1 also modulates signaling of CXCR4, which is activated by stromal-derived factor-1 (SDF-1; Bamidele et al., 2015). Depleting IQGAP1 from leukemic T-cells reduces CXCR4 expression and trafficking to the plasma membrane. The mechanism appears to be that SDF-1 induces IQGAP1 translocation to CXCR4-containing early endosomes (Fig. 3 A). By also binding α-tubulin in microtubules, IQGAP1 coordinates CXCR4 post-endocytic trafficking and recycling to the cell surface. Furthermore, IQGAP1 knockdown impairs SDF-1-stimulated ERK activation and cell migration (Bamidele et al., 2015). This indicates that, similar to RTKs, IQGAP1 modulates MAPK activation downstream of GPCRs. IQGAP1 was also identified as an interactor of the CC chemokine receptor-1 (CCR1) in a mass spectrometry screen (Huttlin et al., 2015), but without further investigation.

### Opioid receptors

The effects of opioids on pain are mediated by the µ-, δ-, κ-opioid, and nociception GPCRs. The μ-opioid receptor MOR1 is the main morphine receptor (Matthes et al., 1996); understanding MOR1 signaling is necessary to reduce the deleterious side effects of morphine. The opioid peptide DAMGO, an alternative agonist of MOR1, augments the amount of cellular IQGAP1 (Civciristov et al., 2019). IQGAP1 knockdown abrogates DAMGO stimulation of both Rac1 and nuclear ERK activities, indicating that IQGAP1 couples DAMGO/MOR1 to GTPase and MAPK signaling (Fig. 3 B). By contrast, morphine stimulation of MOR1 activates ERK and PKCα independently of IQGAP1 (Civciristov et al., 2019; Fig. 3 B). Thus, the specific activating ligand can determine whether IQGAP1 participates in GPCR signaling (termed “biased signaling”; Smith et al., 2018).

The δ-opioid receptor DOR1 is activated by the opioid peptide deltorphin. Analogous to CXCR4, IQGAP1 depletion impairs deltorphin-induced DOR1 intracellular trafficking and signaling to ERK (Bamidele et al., 2015). Whether IQGAP1 binds to DOR1 or functions in signaling of other opioid receptors is unknown.

### Peptide hormone receptors

Consistent with its role in cell motility (Mataraza et al., 2003), IQGAP1 may interact with GPCRs at the leading edge of cells. Activation of the kisspeptin receptor KISS1R by the peptide hormone kisspeptin-10 (KP-10) stimulates invasion of breast carcinoma cells via transactivation of EGFR (Zajac et al., 2011). IQGAP1 binds constitutively to KISS1R; KP-10 does not alter binding (Cvetkovic et al., 2013). Moreover, IQGAP1 and KISS1R colocalize at the leading edge of migrating breast epithelial cells. Importantly, phosphorylation of EGFR by KP-10 is inhibited by IQGAP1 depletion (Cvetkovic et al., 2013), suggesting that IQGAP1 regulates transactivation of EGFR by KISS1R.

The peptide hormone endothelin-1 (ET-1) mediates vasoconstriction via two ET-1 receptors (ET-1Rs), ET_A_R and ET_B_R. Activation of ET-1Rs by ET-1 in ovarian carcinoma cells increases IQGAP1 mRNA and protein levels. ET-1 also promotes interactions between IQGAP1 and β-arrestin1. This complex increases active RhoA and RhoC whereas, by recruiting RacGAP1, it inactivates Rac1 (Chellini et al., 2019; Fig. 3 C). By modulating GTPase signaling, the IQGAP1:β-arrestin1 complex coordinates ET-1/ET-1R-driven cell migration and metastasis. Moreover, IQGAP1:β-arrestin1 promotes invadopodium formation and stimulates secretion and activation of matrix metalloproteinases that degrade the extracellular matrix (ECM), which increases cell invasion (Fig. 3 C). High expression of ET_A_R/IQGAP1/β-arrestin1 positively correlates with poor prognosis in ovarian carcinoma patients (Chellini et al., 2019), suggesting that the IQGAP1:β-arrestin1 interaction could contribute to ovarian carcinoma.

### Lipid receptors

Prostaglandins are fatty acid derivatives produced throughout the human body that mediate diverse actions by binding cognate receptors. Mass spectrometry analysis of the prostaglandin D2 receptor 1 (DP1) interactome identified IQGAP1 (Fréchette et al., 2021). Moreover, IQGAP1 co-immunoprecipitates and colocalizes with DP1. Prostaglandin D2 (PGD_2_) enhances the DP1:IQGAP1 association and redistributes the two proteins to the perinuclear region. IQGAP1 knockdown reduces PGD_2_-induced DP1 internalization and ERK activation (Fréchette et al., 2021). These data suggest that, analogous to CXCR4 and DOR1, IQGAP1 stimulates DP1 trafficking and coupling to MAPK.

The lysophosphatidic acid receptor 1 (LPA1), another lipid receptor, is overexpressed in breast carcinoma and increases metastasis (Liu et al., 2009a). In breast cancer cells, IQGAP1 binds to LPA1 and forms a constitutive complex with β-arrestin2 and Rap1A. LPA stimulates Rap1A and IQGAP1 to associate with LPA1. Interestingly, LPA induces colocalization of IQGAP1 and β-arrestin2 in lamellipodia of migrating cells (Fig. 3 D). Loss of IQGAP1 impairs LPA-stimulated invasion and migration of breast carcinoma cells (Alemayehu et al., 2013). Together, these data indicate that LPA may contribute to breast cancer via IQGAP1, β-arrestin2, and Rap1A.

### Other GPCRs

Leucine-rich repeat-containing G protein–coupled receptors (LGRs) are activated by secreted R-spondins (RSPOs) to induce both canonical (Wnt/β-catenin) and non-canonical (β-catenin-independent) Wnt signaling (Glinka et al., 2011). LGR4, one of the three human LGRs, binds to IQGAP1. RSPO increases binding of IQGAP1 to the Wnt transducer Dvl, and this association is hypothesized to bridge RSPO/LGR4 to the Wnt signalosome (Carmon et al., 2014). IQGAP1 also recruits MEK1/2 to RSPO/LGR4 to phosphorylate LRP6, the co-receptor of the Wnt receptor Frizzled, thereby promoting canonical Wnt/β-catenin signaling. Note that IQGAP1 also regulates canonical Wnt signaling independently of GPCRs: IQGAP1 increases β-catenin nuclear translocation by protecting it from degradation in the cytoplasm (Briggs et al., 2002). In the non-canonical pathway, Wnt and RSPO3 enhance both association of IQGAP1 with the actin regulators N-WASP and mDia1, and its colocalization with LGR4 (Carmon et al., 2014). Thus, IQGAP1 couples LGR4 to both the canonical and non-canonical Wnt pathways.

IQGAP1 also binds constitutively to LGR5 (Carmon et al., 2017), another LGR that potentiates Wnt/β-catenin signaling with both oncogenic and tumor suppressor roles in colorectal carcinoma (Morgan et al., 2018). Overexpression of LGR5 reduces serine phosphorylation of IQGAP1, which enhances its binding to active Rac1 and actin (Carmon et al., 2017; Fig. 3 E). This effect is independent of RSPO. Moreover, depletion of LGR5 or IQGAP1 from colon cancer cells decreases the amount of β-catenin at the plasma membrane and alters the formation of cortical actin. These data, combined with prior observations that Rac1-GTP reduces binding between β-catenin and IQGAP1 in vitro (Fukata et al., 1999), led the authors to speculate that LGR5 overexpression would reduce β-catenin binding to IQGAP1, enhance formation of the E-cadherin:β-catenin:α-catenin complex, and increase actin cross-linking to promote cell–cell adhesion (Carmon et al., 2017; Fig. 3 E).

The orphan G protein–coupled receptor 161 (GPR161) is overexpressed in breast cancer, where it promotes cell migration, proliferation, and invasion. IQGAP1 and β-arrestin2 co-immunoprecipitate with GPR161 from breast cancer cells. IQGAP1 knockdown attenuates GPR161-induced cell proliferation and migration by an unknown mechanism. Because GPR161 and IQGAP1 are both overexpressed in breast cancer (Feigin et al., 2014), their crosstalk may participate in carcinogenesis.

M3-muscarinic acetylcholine receptors (M3-mAChRs) activate MAPK and Ca^2+^ signaling to promote cell proliferation. Stimulating Chinese hamster ovary cells transfected with M3-mAChRs with the muscarinic agonist carbachol promotes translocation of both Rac1 and IQGAP1 to cell junctions where they colocalize (Ruiz-Velasco et al., 2002). M3-mAChR activation increases active Rac1 (Ruiz-Velasco et al., 2002), probably via its interaction with IQGAP1, which stabilizes GTP-bound Rac1 (Mataraza et al., 2003). IQGAP1 also scaffolds actin to Rac1, suggesting that it may mediate M3-mAChR-induced cortical cytoskeleton rearrangements.

### Downstream of GPCRs

In addition to binding β-arrestins, IQGAP1 crosstalks with G-proteins and their regulators to influence GPCR signaling. Regulator of G protein signaling 16 (Rgs16), which is highly expressed in human CD8^+^ tumor-infiltrating lymphocytes (TILs), impairs GPCR signaling by binding G_α_ (Chen et al., 1997). Mass spectrometry analysis of the interactome of tagged Rgs16 in CD8^+^ T-cells identified IQGAP1; the interaction was confirmed by co-immunoprecipitation (Weisshaar et al., 2022). Rgs16 deficiency enhances Ras and B-Raf co-immunoprecipitation with IQGAP1. Moreover, ERK phosphorylation is greater in Rgs16^−/−^ CD8^+^ TILs and T-cell receptor-stimulated T cells than in Rgs16^+/+^ counterparts (Weisshaar et al., 2022). The authors propose that Rgs16 interaction with IQGAP1 inhibits Ras and B-Raf recruitment, thereby impairing ERK activation. Because Rgs16 suppresses CD8^+^ T-cell anti-tumor function by decreasing ERK activation and alters patients’ responses to immune checkpoint inhibition, this mechanism may have potential chemotherapeutic implications.

IQGAP1 also intersects signaling by G_α12_, one of the four sub-families of G_α_ subunits of G proteins. G_α12_ signaling is increased in primary nasopharyngeal carcinoma (NPC) cells (Liu et al., 2009b). Knockdown of G_α12_ reduces IQGAP1 expression. G_α12_ knockdown also impairs migration and invasion of NPC cells and reverses their neoplastic phenotype, while overexpression of IQGAP1 partially suppresses these effects. Interestingly, reducing IQGAP1 in NPC cells elicits effects similar to those produced by G_α12_ knockdown (Liu et al., 2009b). Together, these data raise the possibility that IQGAP1 contributes to tumorigenesis promoted by G_α12_.

## IQGAP1 interacts with other classes of cell surface receptors

Though RTKs and GPCRs constitute the majority of transmembrane receptors with which IQGAP1 interacts, it also associates with receptor serine/threonine kinases (RSTKs), glutamate-gated ion channels (GICs), adhesion receptors, T cell receptors (TCRs), and receptor protein tyrosine phosphatases (RPTPs).

### Receptor serine/threonine kinases: IQGAP1 modulates TGF-β signaling

RSTKs initiate signaling via their intracellular serine/threonine kinase activity (Massagué and Weis-Garcia, 1996). Transforming growth factor-β receptor 2 (TGFβR2) is the only RSTK documented to associate with IQGAP1 (Liu et al., 2013). TGF-β signaling is initiated upon binding of the cytokines TGF-β1, -β2, or -β3 to TGFβR2, leading to the recruitment and activation of TGFβR1. In turn, TGFβR1 activates the SMAD transcription factors to regulate expression of target genes (Vander Ark et al., 2018). TGF-β1 induces the formation of a TGFβR2:IQGAP1 complex in liver pericytes. Once bound, IQGAP1 promotes TGFβR2 degradation by recruiting the ubiquitin ligase SMURF1. By reducing TGFβR2 protein levels, IQGAP1 inhibits TGF-β1-driven differentiation of pericytes into tumor-associated myofibroblasts (Liu et al., 2013), suggesting that the inhibitory TGFβR2:IQGAP1 interaction may constrain tumor growth. A similar mechanism has been suggested in bladder carcinoma cells, where IQGAP1 knockdown increases the amount of TGFβR2 and enhances TGF-β signaling (Hensel et al., 2015). Interestingly, mass spectrometry identified TGFβR1 as a putative interactor of IQGAP1 (Tang et al., 2017), but the observation was not investigated further. TGF-β1 modulates the abundance of IQGAP1. While TGF-β1 decreases the amount of IQGAP1 in hepatic stellate cells (Liu et al., 2013) and lung fibroblasts (Zong et al., 2015), it increases IQGAP1 expression in mammary epithelial cells (Xie et al., 2003), suggesting a cell-specific crosstalk between TGF-β1 and IQGAP1.

### Glutamate-gated ion channels: IQGAP1 coordinates synaptic transmission

Glutamate-gated ion channels (GICs) are cell surface receptors coupled to ion channels that coordinate synaptic transmission in the brain by mediating ion flux on binding the neurotransmitter glutamate (Lemoine et al., 2012). IQGAP1 interacts with two GICs in neurons: the α-amino-3-hydroxy-5-methyl-4-isoxazole propionic acid (AMPA) and N-methyl-D-aspartate (NMDA) receptors. The association of IQGAP1 with the AMPA receptor subunit GluR4 was identified both in a yeast two-hybrid screen and by colocalization in hippocampal neurons (Nuriya et al., 2005). Although not demonstrated, the authors speculate that IQGAP1-mediated cytoskeletal rearrangements promote trafficking of GluR4 to the plasma membrane (Nuriya et al., 2005). In hippocampal cells, IQGAP1 binds to the NMDA receptor subunits NR2A and NR2B, as well as to the scaffold protein PSD-95 that stabilizes NMDA receptors. IQGAP1 promotes targeting of NR2A to the hippocampal plasma membrane in both mice and cells (Gao et al., 2011), similar to the mechanism suggested for GluR4. IQGAP1 augments NMDA-stimulated ERK activation, which regulates histone PTMs to influence expression of genes involved in memory consolidation (Liu et al., 2020). This mechanism could explain the memory defects observed in IQGAP1-null mice (Gao et al., 2011). Interestingly, the Ca^2+^ flux mediated by activated NMDA stimulates binding of IQGAP1 to the microtubule-associated protein Lis1 to drive neuronal motility (Kholmanskikh et al., 2006). Together, these studies indicate that GIC:IQGAP1 complexes coordinate neuronal activities, which may contribute to cognitive physiology and pathology.

### Adhesion receptors: IQGAP1 couples adhesion receptors to cytoskeletal dynamics

**Figure 4.**
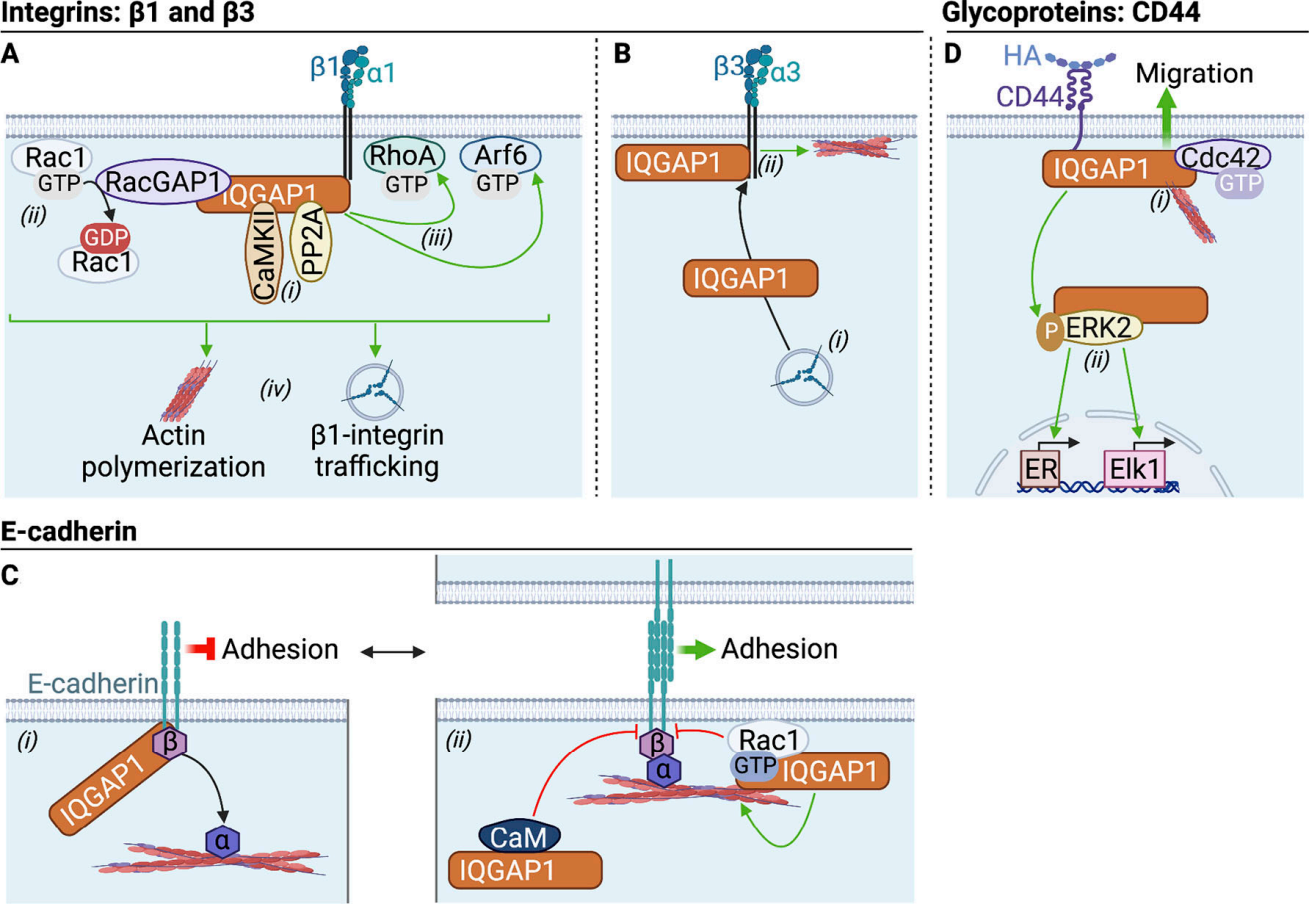
**IQGAP1 participates in adhesion receptor signaling. (A)** β1-integrin: IQGAP1 binds to β1-integrin, (i) together with the kinase CaMKII and the phosphatase PP2A, which assemble and disassemble the complex, respectively. (ii) Rac1 is inactivated by RacGAP1 in the complex, (iii) while RhoA and Arf6 are activated. (iv) These associations promote actin polymerization and influence β1-integrin trafficking. **(B)** β3-integrin: (i) IQGAP1 promotes membrane targeting of β3-integrin. (ii) At the plasma membrane, IQGAP1 interacts with β3-integrin, and the complex enhances cortical actin polymerization. **(C)** E-cadherin: (i) IQGAP1 binds both E-cadherin and β-catenin (β), which dissociates α-catenin (α) from adherens junctions, leading to loosened intercellular adhesion. (ii) Binding of calmodulin (CaM) or active Rac1/Cdc42 (only Rac1 shown) to IQGAP1 prevents its interaction with E-cadherin and β-catenin, thereby stabilizing E-cadherin:β-catenin:α-catenin complexes. Moreover, IQGAP1 bound to Rac1/Cdc42-GTP stimulates actin crosslinking at adherens junctions. **(D)** CD44: Hyaluronan (HA) initiates the formation of a CD44:IQGAP1 complex at the cell surface. (i) IQGAP1 scaffolds actin and active Cdc42 in the complex to stimulate cell migration. (ii) Downstream of HA/CD44, IQGAP1 activates ERK2 which, in turn, increases estrogen receptor (ER) and Elk1 transcriptional activities. Green and red arrows represent stimulation and inhibition, respectively. The figure was generated in BioRender.

Adhesion receptors mediate cell adhesion by binding ligands on the surface of adjacent cells or the ECM. Heterodimeric integrins, composed of α- and β-subunits, are adhesion receptors that commonly bind specific glycoproteins of the ECM (Kechagia et al., 2019). IQGAP1 binds to β1-integrin, together with Rac1. Protein phosphatase 2A (PP2A) and Ca^2+^/calmodulin-dependent protein kinase II (CaMKII) are also in the complex (Fig. 4 A). While PP2A activity promotes the association, activation of CaMKII by EGF dissociates the complex, implying phosphorylation-dependent interactions (Suzuki et al., 2005; Takahashi and Suzuki, 2006). IQGAP1 enhances actin cross-linking by the complex (Nakajima et al., 2005). In mouse embryonic fibroblasts and human osteosarcoma cells, the GTPase activating protein RacGAP1 is also found in the β1-integrin:Rac1:IQGAP1 complex, where it inactivates Rac1 (Jacquemet et al., 2013b). In contrast, β1-integrin:IQGAP1 increases active RhoA (Jacquemet et al., 2013a) and Arf6, which coordinates β1-integrin trafficking (Jacquemet and Humphries, 2013; Fig. 4 A). These findings demonstrate that IQGAP1 couples β1-integrin to actin and small GTPases to modulate adhesion.

IQGAP1 binds β3-integrin in pulmonary VE cells (Bhattacharya et al., 2012). IQGAP1 knockdown decreases trafficking of β3-integrin to cell–cell junctions, which prevents cortical actin formation, ultimately impairing vascular barrier protection (Fig. 4 B). This mechanism could explain the greater pulmonary vascular permeability in IQGAP1-null mice with experimentally induced pneumonia than in wild-type littermates (Bhattacharya et al., 2012). IQGAP1 also binds several integrin-associated proteins, including integrin-linked kinase (Dobreva et al., 2008), α-actinin (Rigothier et al., 2012), and filamin (Jacquemet et al., 2013b). Hence, the functions of IQGAP1 in cell adhesion may also arise from scaffolding integrins with downstream proteins.

The cadherin family are Ca^2+^-dependent adhesion receptors that modulate intercellular adhesion through the assembly/disassembly of adherens junctions (AJs). AJs are formed upon trans-dimerization of classic cadherin receptors on adjacent cells, each bound intracellularly to β- or γ-catenin and α-catenin linked to actin (Yu et al., 2019). IQGAP1 binds to E-cadherin and β-catenin, which triggers the dissociation of α-catenin from AJ structures, thereby loosening intercellular adhesion (Fig. 4 C; Kuroda et al., 1998). This mechanism contributes to cell–cell dissociation during cell scattering (Fukata et al., 2001) and carcinoma cell movement (Shimao et al., 2002). Binding of active Cdc42 or Rac1 to IQGAP1 stabilizes AJs by preventing β-catenin:IQGAP1 interactions, (Fukata et al., 1999), inhibiting E-cadherin endocytosis (Izumi et al., 2004), and stimulating actin crosslinking (Noritake et al., 2004; Fig. 4 C). Similarly, binding of calmodulin to IQGAP1 blocks its interaction with E-cadherin, thereby preventing IQGAP1-mediated AJ disassembly (Li et al., 1999). Cells at the invasive front of colorectal and ovarian tumors have more IQGAP1 staining, especially at sites of intercellular contacts, than central tumor and normal cells, indicating that IQGAP1-mediated AJ disruption may drive tumor invasion (Nabeshima et al., 2002; Dong et al., 2006).

IQGAP1 also interacts with VE-cadherin, which promotes VE-cadherin localization at sites of cell–cell contacts (Yamaoka-Tojo et al., 2006). Like E-cadherin, IQGAP1 binding disassembles the VE-cadherin:β-catenin:α-catenin complex (Yuan et al., 2013). Moreover, by scaffolding VEGFR2 and VE-cadherin, IQGAP1 promotes VEGF-stimulated, ROS-mediated phosphorylation of VE-cadherin, which loosens AJs and increases angiogenesis (Fig. 2 A; Yamaoka-Tojo et al., 2006). In dorso-hippocampal cells, IQGAP1 forms a complex with N-cadherin and ERK2. Reducing N-cadherin:IQGAP1:ERK2 interactions impairs mouse long-term fear memory, implying that IQGAP1 links N-cadherin to ERK signaling to mediate memory consolidation (Schrick et al., 2007). In rat testis, IQGAP1 interacts with N-cadherin and β-catenin, which regulates intercellular adhesion during spermatogenesis (Lui et al., 2005). Importantly, the interaction of IQGAP1 with β-catenin also influences Wnt/β-catenin signaling (for a review, see Smith et al., 2015).

Finally, IQGAP1 participates in cell adhesion via its interaction with the transmembrane glycoprotein CD44, the receptor for hyaluronan (HA; Bourguignon et al., 2005; Skandalis et al., 2010). In ovarian carcinoma cells, HA stimulates CD44:IQGAP1 association. HA also recruits Cdc42 and actin to this complex, which induces cytoskeletal rearrangements and increases tumor cell migration (Fig. 4 D). HA/CD44 also promotes the interaction of IQGAP1 with ERK2. This increases ERK2 nuclear activity, which upregulates the transcriptional activities of Elk-1 and estrogen receptor (Bourguignon et al., 2005; Fig. 4 D). IQGAP1 couples HA/CD44 to cytoskeletal functions in other cells: IQGAP1 promotes HA-induced migration and proliferation of fibroblasts (Kozlova et al., 2012) and CD44-mediated adhesion of glioblastoma cells to HA-rich ECM (Wolf et al., 2020).

### TCRs: IQGAP1 modulates immune responses

In immune cells, IQGAP1 negatively regulates TCR signaling. Upon TCR ligation, actin accumulation at immunological synapses is increased in cells depleted of IQGAP1. This upregulates TCR signaling and increases cytokine production (Gorman et al., 2012). Moreover, T cell stimulation with the OX40 ligand induces IQGAP1 binding to OX40, a T cell costimulatory receptor. IQGAP1-deficient CD4^+^ T cells exhibit increased proliferation and cytokine production following OX40 stimulation, illustrating that IQGAP1 restrains OX40 signaling (Okuyama et al., 2020). Stimulated T cells from IQGAP1-null mice have augmented cytokine production due to upregulation of the transcription factor NFAT (Sharma et al., 2011), again suggesting that IQGAP1 impairs T cell signaling by downregulating cytokine production. Interestingly, experimentally induced brain and spinal cord inflammation is more severe in IQGAP1-null mice than in wild-type littermates (Okuyama et al., 2020), implying that IQGAP1, by negatively regulating T cell signaling, could be implicated in inflammatory disorders.

### Receptor protein tyrosine phosphatases: IQGAP1 interacts with PTPµ

RPTPs modulate protein tyrosine phosphorylation using their intracellular phosphatase activity (Tonks, 2006). Via this mechanism, the RPTP PTPµ regulates cadherin-mediated cell adhesion, neurite outgrowth, and axon guidance (Burden-Gulley and Brady-Kalnay, 1999). IQGAP1 binds directly to PTPµ in lung adenocarcinoma cells. Active Cdc42 and Rac1 promote this interaction, which stimulates actin remodeling and neurite outgrowth (Oblander and Brady-Kalnay, 2010; Phillips-Mason et al., 2006). The adhesion molecules N-cadherin, E-cadherin, and β-catenin are present in the IQGAP1:PTPμ complex, suggesting functions in intercellular adhesion (Phillips-Mason et al., 2006). There is currently no evidence that IQGAP1 is a substrate of PTPμ, though it is possible that PTPμ regulates the small GTPase-dependent functions of IQGAP1 by catalyzing its dephosphorylation.

## IQGAP1 phosphorylation is modulated by receptor signaling

IQGAP1 interacts with various RTKs, and some of them influence its tyrosine phosphorylation. MET is the only RTK documented to directly phosphorylate IQGAP1, and this occurs exclusively on Tyr^1510^ (Hedman et al., 2020). Importantly, phosphorylation of Tyr^1510^ by MET induces the recruitment of SH2-containing proteins to IQGAP1 (Thines et al., 2023). Replacement of Tyr^1510^ of IQGAP1 with non-phosphorylatable Ala impairs HGF/MET-stimulated Akt activation and cell migration, implying that this phosphorylation event influences IQGAP1 functions (Hedman et al., 2020). More indirectly, VEGF/VEGFR2 activates c-Src kinase, which phosphorylates IQGAP1 at an unidentified residue (Meyer et al., 2008); possibly Tyr^1510^ since c-Src overexpression increases phosphorylation of that residue (Hedman et al., 2020). Similarly, EGF activates protein kinase C which catalyzes IQGAP1 phosphorylation on Ser^1441/1443^. IQGAP1 with phosphorylated Ser^1443^ promotes EGFR activation, demonstrating positive feedback (McNulty et al., 2011). EGF also induces phosphorylation of IQGAP1 at Ser^2^ (Huang et al., 2016), while HER2 and PDGF promote tyrosine phosphorylation of IQGAP1 at unknown sites (Kohno et al., 2013; Kratchmarova et al., 2005; Smith et al., 2010). Whether these latter phosphorylation events are directly catalyzed by RTKs remains to be determined. Interestingly, the GPCR LGR5 promotes dephosphorylation of Ser^1441^/^1443^ of IQGAP1 by an unknown mechanism, which stimulates binding and activation of Rac1 (Carmon et al., 2017). The GPCR GPR161 similarly decreases IQGAP1 Ser phosphorylation (Feigin et al., 2014). We suspect that other ligand/receptor systems influence phosphorylation or other PTMs of IQGAP1 to regulate its interactome and signaling functions.

## IQGAP2 and IQGAP3 integrate receptor signaling

Participation of IQGAP2 and IQGAP3 in receptor signaling has recently emerged. To our knowledge, the only receptor to which IQGAP2 and IQGAP3 bind is the GPCR LGR4. While functions of the LGR4:IQGAP2 complex remain unidentified, IQGAP3 promotes RSPO/LGR4 signaling to Wnt/β-catenin, like IQGAP1 (Carmon et al., 2014). Several other studies document that IQGAP2 and IQGAP3 integrate receptor signaling, albeit without demonstrating binding. IQGAP2 inhibits signaling of the GPCR DP1 to ERK activation, whereas IQGAP3, like IQGAP1, promotes coupling of DP1 to ERK (Fréchette et al., 2021). IQGAP2 also inhibits the production of VEGF, which limits VEGFR2 activation and signaling to Akt and restricts angiogenesis in breast cancer cells (Kumar et al., 2022). This observation reinforces the tumor suppressor properties of IQGAP2. In addition, IQGAP2 is an effector of the type-I interferon-α (IFN-α) receptor. IFN-α induces IQGAP2 binding to the NF-κB transcription factor, which promotes expression of IFN-α target genes with antiviral implications (Brisac et al., 2016). In platelets, IQGAP2 functions downstream of protease activated receptors, which are activated by α-thrombin protease. α-thrombin initiates the interaction of IQGAP2 with Arp2/3 and actin to modulate cytoskeletal reorganization (Schmidt et al., 2003).

IQGAP3, like IQGAP1, augments EGF-stimulated activation of EGFR and signaling to ERK, which has been suggested to enhance IQGAP3-driven tumorigenesis (Monteleon et al., 2015; Yang et al., 2014). In nerve growth factor (NGF) signaling, IQGAP3 promotes NGF-induced clustering of the microtubule and actin-binding adenomatous polyposis coli (APC) protein that initiates the formation of cell extensions (Caro-Gonzalez et al., 2012). Additionally, IQGAP3 has been suggested to coordinate zebrafish embryonic development by coupling FGFR1 activation to MAPK signaling (Fang et al., 2015). Opposite to inhibition by IQGAP1, IQGAP3 activates TGF-β signaling in hepatocytes, which drives tumorigenic EMT (Shi et al., 2017).

## Regulation of IQGAP interactions

The interactions of IQGAP proteins with receptors and signaling molecules must be tightly regulated in time and space to avoid disrupted and/or hyperactive signaling. IQGAP1 undergoes PTMs, some of which have been documented to modulate binding of signaling proteins. For example, ubiquitination of Lys^1155^ and Lys^1230^ decreases IQGAP1 binding to Cdc42 and Rac1 (Gorisse et al., 2020), while phosphorylation of IQGAP1 Tyr^1510^ recruits selective SH2-containing proteins (Thines et al., 2023). Interestingly, activation of the RTKs VEGFR2 and PDGFR-β by their cognate ligands induces IQGAP1 phosphorylation and receptor binding (Kohno et al., 2013; Yamaoka-Tojo et al., 2004). This indicates that phosphorylation may promote selected receptor:IQGAP interactions, possibly through specific recruitment at the newly modified residue. PTMs may also influence IQGAP conformation, thereby modulating the accessibility of binding domains to their partners. Additionally, PTMs may induce IQGAP intracellular translocation for interactions with receptors at trafficking vesicles or the plasma membrane.

Other mechanisms could regulate IQGAP scaffolding. Because each IQGAP molecule binds numerous proteins, competition for binding could impair scaffolding. This concept has been experimentally demonstrated: Ca^2+^/calmodulin and EGFR compete for binding to the IQGAP1 IQ domain (McNulty et al., 2011). In contrast, cooperative binding, where binding of one protein enhances binding of another protein, could positively regulate IQGAP scaffolding. For example, ERK1 facilitates MEK binding to IQGAP1 (Roy et al., 2005). IQGAP1 dimerizes (Ren et al., 2005; Liu et al., 2016), which could also influence scaffolding. IQGAPs in dimers could stabilize binding of selected partners, as suggested from the crystal structure where four molecules of GTP-Cdc42 bind two IQGAP2-GRD molecules in a parallel dimer (LeCour et al., 2016). Alternatively, each IQGAP molecule in a dimer could bind different proteins to assemble a functional signalosome. Finally, IQGAP expression, trafficking, and stability, whose regulatory mechanisms remain largely unknown, are likely to determine the formation of specific IQGAP-mediated complexes in selected cell types/tissues.

## Conclusion

The multidomain composition of IQGAP1 enables scaffolding of multiprotein complexes, comprising both receptors and signaling proteins. Thus, IQGAP1 functions as a central platform that couples cell surface receptor activation to intracellular responses. Although less characterized, analogous roles for IQGAP2 and IQGAP3 have recently emerged. The differential functions of the IQGAPs in receptor signaling confirm their non-redundancy and emphasize the importance of studying all three proteins. An important unanswered question is what mechanisms determine dynamic and selective assembly of IQGAP-mediated signalosomes. Further elucidation of the biochemical and cellular properties of IQGAPs, as well as structural characterization of IQGAP complexes, are required to enhance our understanding of regulation of scaffolding.

Importantly, receptor:IQGAP complexes have been identified in several cancer cell lines, with tumorigenic implications. IQGAP-mediated receptor signaling also contributes to other diseases, like diabetes and macular degeneration. Pathogenic signaling may arise from overexpression of IQGAPs and/or receptors, as is commonly observed in neoplastic cells, increasing the number of receptor:IQGAP complexes and amplifying signaling. Alternatively, altered dynamic regulation of the complexes could disturb signaling, disrupting normal cell functions. Elucidating the molecular mechanisms by which receptor:IQGAP complexes contribute to disease could lead to the development of small molecule inhibitor therapeutics which could specifically and selectively target these interactions.

## References

[bib1] Abel, A.M., K.M. Schuldt, K. Rajasekaran, D. Hwang, M.J. Riese, S. Rao, M.S. Thakar, and S. Malarkannan. 2015. IQGAP1: Insights into the function of a molecular puppeteer. Mol. Immunol. 65:336–349. 10.1016/j.molimm.2015.02.012 25733387 PMC4480615

[bib2] Alemayehu, M., M. Dragan, C. Pape, I. Siddiqui, D.B. Sacks, G.M. Di Guglielmo, A.V. Babwah, and M. Bhattacharya. 2013. β-Arrestin2 regulates lysophosphatidic acid-induced human breast tumor cell migration and invasion via Rap1 and IQGAP1. PLoS One. 8:e56174. 10.1371/journal.pone.0056174 23405264 PMC3566084

[bib3] Appert-Collin, A., P. Hubert, G. Crémel, and A. Bennasroune. 2015. Role of ErbB receptors in cancer cell migration and invasion. Front. Pharmacol. 6:283. 10.3389/fphar.2015.00283 26635612 PMC4657385

[bib4] Arienti, C., M. Zanoni, S. Pignatta, A. Del Rio, S. Carloni, M. Tebaldi, G. Tedaldi, and A. Tesei. 2016. Preclinical evidence of multiple mechanisms underlying trastuzumab resistance in gastric cancer. Oncotarget. 7:18424–18439. 10.18632/oncotarget.7575 26919099 PMC4951299

[bib5] Ashino, T., T. Kohno, V. Sudhahar, D. Ash, M. Ushio-Fukai, and T. Fukai. 2018. Copper transporter ATP7A interacts with IQGAP1, a Rac1 binding scaffolding protein: Role in PDGF-induced VSMC migration and vascular remodeling. Am. J. Physiol. Cell Physiol. 315:C850–C862. 10.1152/ajpcell.00230.2018 30257103 PMC6336942

[bib6] Balenci, L., Y. Saoudi, D. Grunwald, J.C. Deloulme, A. Bouron, A. Bernards, and J. Baudier. 2007. IQGAP1 regulates adult neural progenitors in vivo and vascular endothelial growth factor-triggered neural progenitor migration in vitro. J. Neurosci. 27:4716–4724. 10.1523/JNEUROSCI.0830-07.2007 17460084 PMC6672986

[bib7] Bamidele, A.O., K.N. Kremer, P. Hirsova, I.C. Clift, G.J. Gores, D.D. Billadeau, and K.E. Hedin. 2015. IQGAP1 promotes CXCR4 chemokine receptor function and trafficking via EEA-1+ endosomes. J. Cell Biol. 210:257–272. 10.1083/jcb.201411045 26195666 PMC4508899

[bib8] Bañón-Rodríguez, I., M. Gálvez-Santisteban, S. Vergarajauregui, M. Bosch, A. Borreguero-Pascual, and F. Martín-Belmonte. 2014. EGFR controls IQGAP basolateral membrane localization and mitotic spindle orientation during epithelial morphogenesis. EMBO J. 33:129–145. 10.1002/embj.201385946 24421325 PMC3989607

[bib9] Benseñor, L.B., H.-M. Kan, N. Wang, H. Wallrabe, L.A. Davidson, Y. Cai, D.A. Schafer, and G.S. Bloom. 2007. IQGAP1 regulates cell motility by linking growth factor signaling to actin assembly. J. Cell Sci. 120:658–669. 10.1242/jcs.03376 17264147

[bib10] Bhattacharya, M., G. Su, X. Su, J.A. Oses-Prieto, J.T. Li, X. Huang, H. Hernandez, A. Atakilit, A.L. Burlingame, M.A. Matthay, and D. Sheppard. 2012. IQGAP1 is necessary for pulmonary vascular barrier protection in murine acute lung injury and pneumonia. Am. J. Physiol. Lung Cell. Mol. Physiol. 303:L12–L19. 10.1152/ajplung.00375.2011 22561460 PMC3426434

[bib11] Blagoev, B., I. Kratchmarova, S.-E. Ong, M. Nielsen, L.J. Foster, and M. Mann. 2003. A proteomics strategy to elucidate functional protein-protein interactions applied to EGF signaling. Nat. Biotechnol. 21:315–318. 10.1038/nbt790 12577067

[bib12] Bourguignon, L.Y., E. Gilad, K. Rothman, and K. Peyrollier. 2005. Hyaluronan-CD44 interaction with IQGAP1 promotes Cdc42 and ERK signaling, leading to actin binding, Elk-1/estrogen receptor transcriptional activation, and ovarian cancer progression. J. Biol. Chem. 280:11961–11972. 10.1074/jbc.M411985200 15655247

[bib13] Braicu, C., M. Buse, C. Busuioc, R. Drula, D. Gulei, L. Raduly, A. Rusu, A. Irimie, A.G. Atanasov, O. Slaby, . 2019. A comprehensive review on MAPK: A promising therapeutic target in cancer. Cancers. 11:1618. 10.3390/cancers11101618 31652660 PMC6827047

[bib14] Briggs, M.W., and D.B. Sacks. 2003. IQGAP proteins are integral components of cytoskeletal regulation. EMBO Rep. 4:571–574. 10.1038/sj.embor.embor867 12776176 PMC1319206

[bib15] Briggs, M.W., Z. Li, and D.B. Sacks. 2002. IQGAP1-mediated stimulation of transcriptional co-activation by beta-catenin is modulated by calmodulin. J. Biol. Chem. 277:7453–7465. 10.1074/jbc.M104315200 11734550

[bib16] Brisac, C., S. Salloum, V. Yang, E.A.K. Schaefer, J.A. Holmes, S. Chevaliez, J. Hong, C. Carlton-Smith, N. Alatrakchi, A. Kruger, . 2016. IQGAP2 is a novel interferon-alpha antiviral effector gene acting non-conventionally through the NF-κB pathway. J. Hepatol. 65:972–979. 10.1016/j.jhep.2016.06.028 27401546 PMC5656012

[bib17] Bryant, J.A., R.S. Finn, D.J. Slamon, T.F. Cloughesy, and A.C. Charles. 2004. EGF activates intracellular and intercellular calcium signaling by distinct pathways in tumor cells. Cancer Biol. Ther. 3:1243–1249. 10.4161/cbt.3.12.1233 15611621

[bib18] Burden-Gulley, S.M., and S.M. Brady-Kalnay. 1999. PTPmu regulates N-cadherin-dependent neurite outgrowth. J. Cell Biol. 144:1323–1336. 10.1083/jcb.144.6.1323 10087273 PMC2150569

[bib19] Carmon, K.S., X. Gong, J. Yi, A. Thomas, and Q. Liu. 2014. RSPO-LGR4 functions via IQGAP1 to potentiate Wnt signaling. Proc. Natl. Acad. Sci. USA. 111:E1221–E1229. 10.1073/pnas.1323106111 24639526 PMC3977305

[bib20] Carmon, K.S., X. Gong, J. Yi, L. Wu, A. Thomas, C.M. Moore, I. Masuho, D.J. Timson, K.A. Martemyanov, and Q.J. Liu. 2017. LGR5 receptor promotes cell-cell adhesion in stem cells and colon cancer cells via the IQGAP1-Rac1 pathway. J. Biol. Chem. 292:14989–15001. 10.1074/jbc.M117.786798 28739799 PMC5592675

[bib21] Caro-Gonzalez, H.Y., L.N. Nejsum, K.A. Siemers, T.A. Shaler, W.J. Nelson, and A.I. Barth. 2012. Mitogen-activated protein kinase (MAPK/ERK) regulates adenomatous polyposis coli during growth-factor-induced cell extension. J. Cell Sci. 125:1247–1258. 10.1242/jcs.095166 22399805 PMC3324582

[bib22] Casar, B., I. Arozarena, V. Sanz-Moreno, A. Pinto, L. Agudo-Ibáñez, R. Marais, R.E. Lewis, M.T. Berciano, and P. Crespo. 2009. Ras subcellular localization defines extracellular signal-regulated kinase 1 and 2 substrate specificity through distinct utilization of scaffold proteins. Mol. Cell. Biol. 29:1338–1353. 10.1128/MCB.01359-08 19114553 PMC2643815

[bib23] Casteel, D.E., S. Turner, R. Schwappacher, H. Rangaswami, J. Su-Yuo, S. Zhuang, G.R. Boss, and R.B. Pilz. 2012. Rho isoform-specific interaction with IQGAP1 promotes breast cancer cell proliferation and migration. J. Biol. Chem. 287:38367–38378. 10.1074/jbc.M112.377499 22992742 PMC3488105

[bib24] Chawla, B., A.C. Hedman, S. Sayedyahossein, H.H. Erdemir, Z. Li, and D.B. Sacks. 2017. Absence of IQGAP1 protein leads to insulin resistance. J. Biol. Chem. 292:3273–3289. 10.1074/jbc.M116.752642 28082684 PMC5336162

[bib25] Chellini, L., V. Caprara, F. Spadaro, R. Sestito, A. Bagnato, and L. Rosanò. 2019. Regulation of extracellular matrix degradation and metastatic spread by IQGAP1 through endothelin-1 receptor signalling in ovarian cancer. Matrix Biol. 81:17–33. 10.1016/j.matbio.2018.10.005 30367951

[bib26] Chen, C., B. Zheng, J. Han, and S.C. Lin. 1997. Characterization of a novel mammalian RGS protein that binds to Galpha proteins and inhibits pheromone signaling in yeast. J. Biol. Chem. 272:8679–8685. 10.1074/jbc.272.13.8679 9079700

[bib27] Chen, F., H.-H. Zhu, L.-F. Zhou, S.-S. Wu, J. Wang, and Z. Chen. 2010. IQGAP1 is overexpressed in hepatocellular carcinoma and promotes cell proliferation by Akt activation. Exp. Mol. Med. 42:477–483. 10.3858/emm.2010.42.7.049 20530982 PMC2912475

[bib28] Chen, P.H., X. Chen, and X. He. 2013. Platelet-derived growth factors and their receptors: Structural and functional perspectives. Biochim. Biophys. Acta. 1834:2176–2186. 10.1016/j.bbapap.2012.10.015 23137658 PMC3612563

[bib29] Chen, M., S. Choi, O. Jung, T. Wen, C. Baum, N. Thapa, P.F. Lambert, A.C. Rapraeger, and R.A. Anderson. 2019. The specificity of EGF-stimulated IQGAP1 scaffold towards the PI3K-Akt pathway is defined by the IQ3 motif. Sci. Rep. 9:9126. 10.1038/s41598-019-45671-5 31235839 PMC6591252

[bib30] Chen, Y., J. Mei, P. Zhang, J. Liu, L. Chen, L. Wu, and Y. Zhang. 2022. IQGAP1 is positively correlated with PD-L1 and regulates its expression via mediating STAT proteins phosphorylation. Int. Immunopharmacol. 108:108897. 10.1016/j.intimp.2022.108897 35729832

[bib31] Choi, S., N. Thapa, A.C. Hedman, Z. Li, D.B. Sacks, and R.A. Anderson. 2013. IQGAP1 is a novel phosphatidylinositol 4,5 bisphosphate effector in regulation of directional cell migration. EMBO J. 32:2617–2630. 10.1038/emboj.2013.191 23982733 PMC3791370

[bib32] Choi, S., A.C. Hedman, S. Sayedyahossein, N. Thapa, D.B. Sacks, and R.A. Anderson. 2016. Agonist-stimulated phosphatidylinositol-3,4,5-trisphosphate generation by scaffolded phosphoinositide kinases. Nat. Cell Biol. 18:1324–1335. 10.1038/ncb3441 27870828 PMC5679705

[bib33] Civciristov, S., C. Huang, B. Liu, E.A. Marquez, A.B. Gondin, R.B. Schittenhelm, A.M. Ellisdon, M. Canals, and M.L. Halls. 2019. Ligand-dependent spatiotemporal signaling profiles of the μ-opioid receptor are controlled by distinct protein-interaction networks. J. Biol. Chem. 294:16198–16213. 10.1074/jbc.RA119.008685 31515267 PMC6827304

[bib34] Colavito, S.A. 2020. AXL as a target in breast cancer therapy. J. Oncol. 2020:5291952. 10.1155/2020/5291952 32148495 PMC7042526

[bib35] Cvetkovic, D., M. Dragan, S.J. Leith, Z.M. Mir, H.S. Leong, M. Pampillo, J.D. Lewis, A.V. Babwah, and M. Bhattacharya. 2013. KISS1R induces invasiveness of estrogen receptor-negative human mammary epithelial and breast cancer cells. Endocrinology. 154:1999–2014. 10.1210/en.2012-2164 23525242

[bib36] David, S., C.C. Ghosh, A. Mukherjee, and S.M. Parikh. 2011. Angiopoietin-1 requires IQ domain GTPase-activating protein 1 to activate Rac1 and promote endothelial barrier defense. Arterioscler. Thromb. Vasc. Biol. 31:2643–2652. 10.1161/ATVBAHA.111.233189 21885850 PMC3249617

[bib37] Delgado, E.R., H.L. Erickson, J. Tao, S.P. Monga, A.W. Duncan, and S. Anakk. 2021. Scaffolding protein IQGAP1 is dispensable, but its overexpression promotes hepatocellular carcinoma via YAP1 signaling. Mol. Cell. Biol. 41:e005966-20. 10.1128/MCB.00596-20 PMC8088129 33526450

[bib38] Desole, C., S. Gallo, A. Vitacolonna, F. Montarolo, A. Bertolotto, D. Vivien, P. Comoglio, and T. Crepaldi. 2021. HGF and MET: From brain development to neurological disorders. Front. Cell Dev. Biol. 9:683609. 10.3389/fcell.2021.683609 34179015 PMC8220160

[bib39] Dobreva, I., A. Fielding, L.J. Foster, and S. Dedhar. 2008. Mapping the integrin-linked kinase interactome using SILAC. J. Proteome Res. 7:1740–1749. 10.1021/pr700852r 18327965

[bib40] Dong, P., K. Nabeshima, N. Nishimura, T. Kawakami, T. Hachisuga, T. Kawarabayashi, and H. Iwasaki. 2006. Overexpression and diffuse expression pattern of IQGAP1 at invasion fronts are independent prognostic parameters in ovarian carcinomas. Cancer Lett. 243:120–127. 10.1016/j.canlet.2005.11.024 16387427

[bib41] Erickson, J.W., R.A. Cerione, and M.J. Hart. 1997. Identification of an actin cytoskeletal complex that includes IQGAP and the Cdc42 GTPase. J. Biol. Chem. 272:24443–24447. 10.1074/jbc.272.39.24443 9305904

[bib42] Fang, X., B. Zhang, B. Thisse, G.S. Bloom, and C. Thisse. 2015. IQGAP3 is essential for cell proliferation and motility during zebrafish embryonic development. Cytoskeleton. 72:422–433. 10.1002/cm.21237 26286209 PMC4600665

[bib43] Feigin, M.E., B. Xue, M.C. Hammell, and S.K. Muthuswamy. 2014. G-protein-coupled receptor GPR161 is overexpressed in breast cancer and is a promoter of cell proliferation and invasion. Proc. Natl. Acad. Sci. USA. 111:4191–4196. 10.1073/pnas.1320239111 24599592 PMC3964064

[bib44] Fram, S., H. King, D.B. Sacks, and C.M. Wells. 2014. A PAK6-IQGAP1 complex promotes disassembly of cell-cell adhesions. Cell. Mol. Life Sci. 71:2759–2773. 10.1007/s00018-013-1528-5 24352566 PMC4059965

[bib45] Fréchette, L., J. Degrandmaison, C. Binda, M. Boisvert, L. Côté, T. Michaud, M.-P. Lalumière, L. Gendron, and J.-L. Parent. 2021. Identification of the interactome of the DP1 receptor for Prostaglandin D_2_: Regulation of DP1 receptor signaling and trafficking by IQGAP1. Biochim. Biophys. Acta Gen. Subj. 1865:129969. 10.1016/j.bbagen.2021.129969 34352343

[bib46] Fukata, M., S. Kuroda, M. Nakagawa, A. Kawajiri, N. Itoh, I. Shoji, Y. Matsuura, S. Yonehara, H. Fujisawa, A. Kikuchi, and K. Kaibuchi. 1999. Cdc42 and Rac1 regulate the interaction of IQGAP1 with beta-catenin. J. Biol. Chem. 274:26044–26050. 10.1074/jbc.274.37.26044 10473551

[bib47] Fukata, M., M. Nakagawa, N. Itoh, A. Kawajiri, M. Yamaga, S. Kuroda, and K. Kaibuchi. 2001. Involvement of IQGAP1, an effector of Rac1 and Cdc42 GTPases, in cell-cell dissociation during cell scattering. Mol. Cell. Biol. 21:2165–2183. 10.1128/MCB.21.6.2165-2183.2001 11238950 PMC86844

[bib48] Gao, C., S.F. Frausto, A.L. Guedea, N.C. Tronson, V. Jovasevic, K. Leaderbrand, K.A. Corcoran, Y.F. Guzmán, G.T. Swanson, and J. Radulovic. 2011. IQGAP1 regulates NR2A signaling, spine density, and cognitive processes. J. Neurosci. 31:8533–8542. 10.1523/JNEUROSCI.1300-11.2011 21653857 PMC3121195

[bib49] Glinka, A., C. Dolde, N. Kirsch, Y.L. Huang, O. Kazanskaya, D. Ingelfinger, M. Boutros, C.M. Cruciat, and C. Niehrs. 2011. LGR4 and LGR5 are R-spondin receptors mediating Wnt/β-catenin and Wnt/PCP signalling. EMBO Rep. 12:1055–1061. 10.1038/embor.2011.175 21909076 PMC3185347

[bib50] Gorisse, L., Z. Li, A.C. Hedman, and D.B. Sacks. 2018. IQGAP1 binds the Axl receptor kinase and inhibits its signaling. Biochem. J. 475:3073–3086. 10.1042/BCJ20180594 30185434

[bib51] Gorisse, L., Z. Li, C.D. Wagner, D.K. Worthylake, F. Zappacosta, A.C. Hedman, R.S. Annan, and D.B. Sacks. 2020. Ubiquitination of the scaffold protein IQGAP1 diminishes its interaction with and activation of the Rho GTPase CDC42. J. Biol. Chem. 295:4822–4835. 10.1074/jbc.RA119.011491 32094223 PMC7152761

[bib52] Gorman, J.A., A. Babich, C.J. Dick, R.A. Schoon, A. Koenig, T.S. Gomez, J.K. Burkhardt, and D.D. Billadeau. 2012. The cytoskeletal adaptor protein IQGAP1 regulates TCR-mediated signaling and filamentous actin dynamics. J. Immunol. 188:6135–6144. 10.4049/jimmunol.1103487 22573807 PMC3370139

[bib53] Goto, T., A. Sato, S. Adachi, S. Iemura, T. Natsume, and H. Shibuya. 2013. IQGAP1 protein regulates nuclear localization of β-catenin via importin-β5 protein in Wnt signaling. J. Biol. Chem. 288:36351–36360. 10.1074/jbc.M113.520528 24196961 PMC3868749

[bib54] Groth, C., and M. Lardelli. 2002. The structure and function of vertebrate fibroblast growth factor receptor 1. Int. J. Dev. Biol. 46:393–400. 12141425

[bib55] Gurevich, V.V., and E.V. Gurevich. 2019. GPCR signaling regulation: The role of GRKs and arrestins. Front. Pharmacol. 10:125. 10.3389/fphar.2019.00125 30837883 PMC6389790

[bib56] Hansson, B., B. Morén, C. Fryklund, L. Vliex, S. Wasserstrom, S. Albinsson, K. Berger, and K.G. Stenkula. 2019. Adipose cell size changes are associated with a drastic actin remodeling. Sci. Rep. 9:12941. 10.1038/s41598-019-49418-0 31506540 PMC6736966

[bib57] Hauser, A.S., M.M. Attwood, M. Rask-Andersen, H.B. Schiöth, and D.E. Gloriam. 2017. Trends in GPCR drug discovery: New agents, targets and indications. Nat. Rev. Drug Discov. 16:829–842. 10.1038/nrd.2017.178 29075003 PMC6882681

[bib58] Hayashi, H., K. Nabeshima, M. Aoki, M. Hamasaki, S. Enatsu, Y. Yamauchi, Y. Yamashita, and H. Iwasaki. 2010. Overexpression of IQGAP1 in advanced colorectal cancer correlates with poor prognosis-critical role in tumor invasion. Int. J. Cancer. 126:2563–2574. 10.1002/ijc.24987 19856315

[bib59] Hedman, A.C., J.M. Smith, and D.B. Sacks. 2015. The biology of IQGAP proteins: Beyond the cytoskeleton. EMBO Rep. 16:427–446. 10.15252/embr.201439834 25722290 PMC4388610

[bib60] Hedman, A.C., D.E. McNulty, Z. Li, L. Gorisse, R.S. Annan, and D.B. Sacks. 2020. Tyrosine phosphorylation of the scaffold protein IQGAP1 in the MET pathway alters function. J. Biol. Chem. 295:18105–18121. 10.1074/jbc.RA120.01589133087447

[bib61] Hedman, A.C., Z. Li, L. Gorisse, S. Parvathaneni, C.J. Morgan, and D.B. Sacks. 2021. IQGAP1 binds AMPK and is required for maximum AMPK activation. J. Biol. Chem. 296:100075. 10.1074/jbc.RA120.016193 33191271 PMC7948462

[bib62] Hensel, J., J.E. Duex, C. Owens, G.M. Dancik, M.G. Edwards, H.F. Frierson, and D. Theodorescu. 2015. Patient mutation directed shRNA screen uncovers novel bladder tumor growth suppressors. Cell Growth Differ. 13:1306–1315. 10.1158/1541-7786.MCR-15-0130 PMC4573363 26078295

[bib63] Hilger, D., M. Masureel, and B.K. Kobilka. 2018. Structure and dynamics of GPCR signaling complexes. Nat. Struct. Mol. Biol. 25:4–12. 10.1038/s41594-017-0011-7 29323277 PMC6535338

[bib64] Hu, B., B. Shi, M.J. Jarzynka, J.J. Yiin, C. D’Souza-Schorey, and S.Y. Cheng. 2009. ADP-ribosylation factor 6 regulates glioma cell invasion through the IQ-domain GTPase-activating protein 1-Rac1-mediated pathway. Cancer Res. 69:794–801. 10.1158/0008-5472.CAN-08-2110 19155310 PMC2633432

[bib65] Hu, H.-F., W.W. Xu, Y.-J. Li, Y. He, W.-X. Zhang, L. Liao, Q.-H. Zhang, L. Han, X.-F. Yin, X.-X. Zhao, . 2021. Anti-allergic drug azelastine suppresses colon tumorigenesis by directly targeting ARF1 to inhibit IQGAP1-ERK-Drp1-mediated mitochondrial fission. Theranostics. 11:1828–1844. 10.7150/thno.48698 33408784 PMC7778598

[bib66] Hu, X., J. Li, M. Fu, X. Zhao, and W. Wang. 2021. The JAK/STAT signaling pathway: From bench to clinic. Signal Transduct. Target. Ther. 6:402. 10.1038/s41392-021-00791-1 34824210 PMC8617206

[bib67] Huang, H., M. Haar Petersen, M. Ibañez-Vea, P.S. Lassen, M.R. Larsen, and G. Palmisano. 2016. Simultaneous enrichment of cysteine-containing peptides and phosphopeptides using a cysteine-specific phosphonate adaptable tag (CysPAT) in combination with titanium dioxide (TiO2) chromatography. Mol. Cell. Proteomics. 15:3282–3296. 10.1074/mcp.M115.054551 27281782 PMC5054350

[bib68] Hubbard, S.R., and W.T. Miller. 2007. Receptor tyrosine kinases: Mechanisms of activation and signaling. Curr. Opin. Cell Biol. 19:117–123. 10.1016/j.ceb.2007.02.010 17306972 PMC2536775

[bib69] Huttlin, E.L., L. Ting, R.J. Bruckner, F. Gebreab, M.P. Gygi, J. Szpyt, S. Tam, G. Zarraga, G. Colby, K. Baltier, . 2015. The BioPlex network: A systematic exploration of the human interactome. Cell. 162:425–440. 10.1016/j.cell.2015.06.043 26186194 PMC4617211

[bib70] Ikeda, S., M. Yamaoka-Tojo, L. Hilenski, N.A. Patrushev, G.M. Anwar, M.T. Quinn, and M. Ushio-Fukai. 2005. IQGAP1 regulates reactive oxygen species-dependent endothelial cell migration through interacting with Nox2. Arterioscler. Thromb. Vasc. Biol. 25:2295–2300. 10.1161/01.ATV.0000187472.55437.af 16179592

[bib71] Izumi, G., T. Sakisaka, T. Baba, S. Tanaka, K. Morimoto, and Y. Takai. 2004. Endocytosis of E-cadherin regulated by Rac and Cdc42 small G proteins through IQGAP1 and actin filaments. J. Cell Biol. 166:237–248. 10.1083/jcb.200401078 15263019 PMC2172308

[bib72] Jacquemet, G., and M.J. Humphries. 2013. IQGAP1 is a key node within the small GTPase network. Small GTPases. 4:199–207. 10.4161/sgtp.27451 24355937 PMC4011815

[bib73] Jacquemet, G., D.M. Green, R.E. Bridgewater, A. von Kriegsheim, M.J. Humphries, J.C. Norman, and P.T. Caswell. 2013a. RCP-driven α5β1 recycling suppresses Rac and promotes RhoA activity via the RacGAP1-IQGAP1 complex. J. Cell Biol. 202:917–935. 10.1083/jcb.201302041 24019536 PMC3776348

[bib74] Jacquemet, G., M.R. Morgan, A. Byron, J.D. Humphries, C.K. Choi, C.S. Chen, P.T. Caswell, and M.J. Humphries. 2013b. Rac1 is deactivated at integrin activation sites through an IQGAP1-filamin-A-RacGAP1 pathway. J. Cell Sci. 126:4121–4135. 23843620 10.1242/jcs.121988 PMC3772387

[bib75] Jameson, K.L., P.K. Mazur, A.M. Zehnder, J. Zhang, B. Zarnegar, J. Sage, and P.A. Khavari. 2013. IQGAP1 scaffold-kinase interaction blockade selectively targets RAS-MAP kinase-driven tumors. Nat. Med. 19:626–630. 10.1038/nm.3165 23603816 PMC4190012

[bib76] Jeong, H.-W., Z. Li, M.D. Brown, and D.B. Sacks. 2007. IQGAP1 binds Rap1 and modulates its activity. J. Biol. Chem. 282:20752–20762. 10.1074/jbc.M700487200 17517894

[bib77] Jiang, N., Q. Dai, X. Su, J. Fu, X. Feng, and J. Peng. 2020. Role of PI3K/AKT pathway in cancer: The framework of malignant behavior. Mol. Biol. Rep. 47:4587–4629. 10.1007/s11033-020-05435-1 32333246 PMC7295848

[bib78] Jufvas, Å., M.R. Rajan, C. Jönsson, P. Strålfors, and M.V. Turkina. 2016. Scaffolding protein IQGAP1: An insulin-dependent link between caveolae and the cytoskeleton in primary human adipocytes? Biochem. J. 473:3177–3188. 10.1042/BCJ20160581 27458251

[bib79] Kaplan, N., N. Urao, E. Furuta, S.-J. Kim, M. Razvi, Y. Nakamura, R.D. McKinney, L.B. Poole, T. Fukai, and M. Ushio-Fukai. 2011. Localized cysteine sulfenic acid formation by vascular endothelial growth factor: Role in endothelial cell migration and angiogenesis. Free Radic. Res. 45:1124–1135. 10.3109/10715762.2011.602073 21740309 PMC3685280

[bib80] Kechagia, J.Z., J. Ivaska, and P. Roca-Cusachs. 2019. Integrins as biomechanical sensors of the microenvironment. Nat. Rev. Mol. Cell Biol. 20:457–473. 10.1038/s41580-019-0134-2 31182865

[bib81] Kholmanskikh, S.S., H.B. Koeller, A. Wynshaw-Boris, T. Gomez, P.C. Letourneau, and M.E. Ross. 2006. Calcium-dependent interaction of Lis1 with IQGAP1 and Cdc42 promotes neuronal motility. Nat. Neurosci. 9:50–57. 10.1038/nn1619 16369480

[bib82] Kimura, T., M. Yamaoka, S. Taniguchi, M. Okamoto, M. Takei, T. Ando, A. Iwamatsu, T. Watanabe, K. Kaibuchi, T. Ishizaki, and I. Niki. 2013. Activated Cdc42-bound IQGAP1 determines the cellular endocytic site. Mol. Cell. Biol. 33:4834–4843. 10.1128/MCB.00895-13 24100016 PMC3889562

[bib83] Kohno, T., N. Urao, T. Ashino, V. Sudhahar, H. Inomata, M. Yamaoka-Tojo, R.D. McKinney, T. Fukai, and M. Ushio-Fukai. 2013. IQGAP1 links PDGF receptor-β signal to focal adhesions involved in vascular smooth muscle cell migration: Role in neointimal formation after vascular injury. Am. J. Physiol. Cell Physiol. 305:C591–C600. 10.1152/ajpcell.00011.2013 23657573 PMC3761176

[bib84] Kozlova, I., A. Ruusala, O. Voytyuk, S.S. Skandalis, and P. Heldin. 2012. IQGAP1 regulates hyaluronan-mediated fibroblast motility and proliferation. Cell. Signal. 24:1856–1862. 10.1016/j.cellsig.2012.05.013 22634185

[bib85] Kratchmarova, I., B. Blagoev, M. Haack-Sorensen, M. Kassem, and M. Mann. 2005. Mechanism of divergent growth factor effects in mesenchymal stem cell differentiation. Science. 308:1472–1477. 10.1126/science.1107627 15933201

[bib86] Kumar, D., S.A. Patel, R. Khan, S. Chawla, N. Mohapatra, and M. Dixit. 2022. IQ motif-containing GTPase-activating protein 2 inhibits breast cancer angiogenesis by suppressing VEGFR2-AKT signaling. Mol. Cancer Res. 20:77–91. 10.1158/1541-7786.MCR-20-1044 34615693

[bib87] Kuroda, S., M. Fukata, K. Kobayashi, M. Nakafuku, N. Nomura, A. Iwamatsu, and K. Kaibuchi. 1996. Identification of IQGAP as a putative target for the small GTPases, Cdc42 and Rac1. J. Biol. Chem. 271:23363–23367. 10.1074/jbc.271.38.23363 8798539

[bib88] Kuroda, S., M. Fukata, M. Nakagawa, K. Fujii, T. Nakamura, T. Ookubo, I. Izawa, T. Nagase, N. Nomura, H. Tani, . 1998. Role of IQGAP1, a target of the small GTPases Cdc42 and Rac1, in regulation of E-cadherin- mediated cell-cell adhesion. Science. 281:832–835. 10.1126/science.281.5378.832 9694656

[bib89] LeCour, L., Jr, V.K. Boyapati, J. Liu, Z. Li, D.B. Sacks, and D.K. Worthylake. 2016. The structural basis for Cdc42-induced dimerization of IQGAPs. Structure. 24:1499–1508. 10.1016/j.str.2016.06.016 27524202 PMC5014685

[bib90] Lemoine, D., R. Jiang, A. Taly, T. Chataigneau, A. Specht, and T. Grutter. 2012. Ligand-gated ion channels: New insights into neurological disorders and ligand recognition. Chem. Rev. 112:6285–6318. 10.1021/cr3000829 22988962

[bib91] Li, Z., and D.B. Sacks. 2003. Elucidation of the interaction of calmodulin with the IQ motifs of IQGAP1. J. Biol. Chem. 278:4347–4352. 10.1074/jbc.M208579200 12446675

[bib92] Li, Z., S.H. Kim, J.M.G. Higgins, M.B. Brenner, and D.B. Sacks. 1999. IQGAP1 and calmodulin modulate E-cadherin function. J. Biol. Chem. 274:37885–37892. 10.1074/jbc.274.53.37885 10608854

[bib93] Li, C.H., X.J. Sun, S.S. Niu, C.Y. Yang, Y.P. Hao, J.T. Kou, X.Z. Li, and X.X. Wang. 2018. Overexpression of IQGAP1 promotes the angiogenesis of esophageal squamous cell carcinoma through the AKT and ERK-mediated VEGF-VEGFR2 signaling pathway. Oncol. Rep. 40:1795–1802. 10.3892/or.2018.6558 30015941

[bib94] Liu, S., M. Umezu-Goto, M. Murph, Y. Lu, W. Liu, F. Zhang, S. Yu, L.C. Stephens, X. Cui, G. Murrow, . 2009a. Expression of autotaxin and lysophosphatidic acid receptors increases mammary tumorigenesis, invasion, and metastases. Cancer Cell. 15:539–550. 10.1016/j.ccr.2009.03.027 19477432 PMC4157573

[bib95] Liu, S.C., Y.M. Jen, S.S. Jiang, J.L. Chang, C.A. Hsiung, C.H. Wang, and J.L. Juang. 2009b. G(α)12-mediated pathway promotes invasiveness of nasopharyngeal carcinoma by modulating actin cytoskeleton reorganization. Cancer Res. 69:6122–6130. 10.1158/0008-5472.CAN-08-3435 19602597

[bib96] Liu, C., D.D. Billadeau, H. Abdelhakim, E. Leof, K. Kaibuchi, C. Bernabeu, G.S. Bloom, L. Yang, L. Boardman, V.H. Shah, and N. Kang. 2013. IQGAP1 suppresses TβRII-mediated myofibroblastic activation and metastatic growth in liver. J. Clin. Invest. 123:1138–1156. 10.1172/JCI63836 23454766 PMC3582119

[bib97] Liu, J., V.B. Kurella, L. LeCour Jr, T. Vanagunas, and D.K. Worthylake. 2016. The IQGAP1 N-terminus forms dimers, and the dimer interface is required for binding F-actin and calcium-bound calmodulin. Biochemistry. 55:6433–6444. 10.1021/acs.biochem.6b00745 27798963 PMC5543714

[bib98] Liu, J., X. Ni, Y. Li, M. Chen, W. Chen, Y. Wu, B. Chen, Y. Wu, and M. Xu. 2019. Downregulation of IQGAP1 inhibits epithelial-mesenchymal transition via the HIF1α/VEGF-A signaling pathway in gastric cancer. J. Cell. Biochem. 120:15790–15799. 10.1002/jcb.28849 31090961

[bib99] Liu, X.-Y., B. Yao, J.-R. Hao, L. Jin, Y. Gao, X. Yang, L. Liu, X.-Y. Sun, N. Sun, and C. Gao. 2020. IQGAP1/ERK regulates fear memory formation via histone posttranslational modifications induced by HDAC2. Neurobiol. Learn. Mem. 171:107210. 10.1016/j.nlm.2020.107210 32145408

[bib100] Lui, W.Y., D.D. Mruk, and C.Y. Cheng. 2005. Interactions among IQGAP1, Cdc42, and the cadherin/catenin protein complex regulate Sertoli-germ cell adherens junction dynamics in the testis. J. Cell. Physiol. 202:49–66. 10.1002/jcp.20098 15389538

[bib101] Ma, Y., Z. Jin, J. Huang, S. Zhou, H. Ye, S. Jiang, and K. Yu. 2013. IQGAP1 plays an important role in the cell proliferation of multiple myeloma via the MAP kinase (ERK) pathway. Oncol. Rep. 30:3032–3038. 10.3892/or.2013.2785 24101133

[bib102] Massagué, J., and F. Weis-Garcia. 1996. Serine/threonine kinase receptors: Mediators of transforming growth factor beta family signals. Cancer Surv. 27:41–64. 8909794

[bib103] Mataraza, J.M., M.W. Briggs, Z. Li, A. Entwistle, A.J. Ridley, and D.B. Sacks. 2003. IQGAP1 promotes cell motility and invasion. J. Biol. Chem. 278:41237–41245. 10.1074/jbc.M304838200 12900413

[bib104] Matthes, H.W., R. Maldonado, F. Simonin, O. Valverde, S. Slowe, I. Kitchen, K. Befort, A. Dierich, M. Le Meur, P. Dollé, . 1996. Loss of morphine-induced analgesia, reward effect and withdrawal symptoms in mice lacking the mu-opioid-receptor gene. Nature. 383:819–823. 10.1038/383819a0 8893006

[bib105] McNulty, D.E., Z. Li, C.D. White, D.B. Sacks, and R.S. Annan. 2011. MAPK scaffold IQGAP1 binds the EGF receptor and modulates its activation. J. Biol. Chem. 286:15010–15021. 10.1074/jbc.M111.227694 21349850 PMC3083173

[bib106] Meng, X., O. Krokhin, K. Cheng, W. Ens, and J.A. Wilkins. 2007. Characterization of IQGAP1-containing complexes in NK-like cells: Evidence for rac 2 and RACK1 association during homotypic adhesion. J. Proteome Res. 6:744–750. 10.1021/pr060382t 17269730

[bib107] Meyer, R.D., D.B. Sacks, and N. Rahimi. 2008. IQGAP1-dependent signaling pathway regulates endothelial cell proliferation and angiogenesis. PLoS One. 3:e3848. 10.1371/journal.pone.0003848 19050761 PMC2585478

[bib108] Monteleon, C.L., A. McNeal, E.K. Duperret, S.J. Oh, E. Schapira, and T.W. Ridky. 2015. IQGAP1 and IQGAP3 serve individually essential roles in normal epidermal homeostasis and tumor progression. J. Invest. Dermatol. 135:2258–2265. 10.1038/jid.2015.140 25848980 PMC4537348

[bib109] Morgan, R.G., E. Mortensson, D.N. Legge, B. Gupta, T.J. Collard, A. Greenhough, and A.C. Williams. 2018. LGR5 expression is regulated by EGF in early colorectal adenomas and governs EGFR inhibitor sensitivity. Br. J. Cancer. 118:558–565. 10.1038/bjc.2017.412 29149105 PMC5830587

[bib110] Morgan, C.J., A.C. Hedman, Z. Li, and D.B. Sacks. 2019. Endogenous IQGAP1 and IQGAP3 do not functionally interact with Ras. Sci. Rep. 9:11057. 10.1038/s41598-019-46677-9 31363101 PMC6667474

[bib111] Nabeshima, K., Y. Shimao, T. Inoue, and M. Koono. 2002. Immunohistochemical analysis of IQGAP1 expression in human colorectal carcinomas: Its overexpression in carcinomas and association with invasion fronts. Cancer Lett. 176:101–109. 10.1016/S0304-3835(01)00742-X 11790459

[bib112] Naidu, S., L. Shi, P. Magee, J.D. Middleton, A. Laganá, S. Sahoo, H.S. Leong, M. Galvin, K. Frese, C. Dive, . 2017. PDGFR-modulated miR-23b cluster and miR-125a-5p suppress lung tumorigenesis by targeting multiple components of KRAS and NF-kB pathways. Sci. Rep. 7:15441. 10.1038/s41598-017-14843-6 29133857 PMC5684387

[bib113] Nakajima, E., K. Suzuki, and K. Takahashi. 2005. Mitotic dissociation of IQGAP1 from Rac-bound beta1-integrin is mediated by protein phosphatase 2A. Biochem. Biophys. Res. Commun. 326:249–253. 10.1016/j.bbrc.2004.11.023 15567178

[bib114] Neel, N.F., J. Sai, A.J. Ham, T. Sobolik-Delmaire, R.L. Mernaugh, and A. Richmond. 2011. IQGAP1 is a novel CXCR2-interacting protein and essential component of the “chemosynapse”. PLoS One. 6:e23813. 10.1371/journal.pone.0023813 21876773 PMC3158102

[bib115] Neudauer, C.L., G. Joberty, N. Tatsis, and I.G. Macara. 1998. Distinct cellular effects and interactions of the Rho-family GTPase TC10. Curr. Biol. 8:1151–1160. 10.1016/S0960-9822(07)00486-1 9799731

[bib116] Noritake, J., M. Fukata, K. Sato, M. Nakagawa, T. Watanabe, N. Izumi, S. Wang, Y. Fukata, and K. Kaibuchi. 2004. Positive role of IQGAP1, an effector of Rac1, in actin-meshwork formation at sites of cell-cell contact. Mol. Biol. Cell. 15:1065–1076. 10.1091/mbc.e03-08-0582 14699063 PMC363077

[bib117] Noritake, J., T. Watanabe, K. Sato, S. Wang, and K. Kaibuchi. 2005. IQGAP1: A key regulator of adhesion and migration. J. Cell Sci. 118:2085–2092. 10.1242/jcs.02379 15890984

[bib118] Nuriya, M., S. Oh, and R.L. Huganir. 2005. Phosphorylation-dependent interactions of alpha-Actinin-1/IQGAP1 with the AMPA receptor subunit GluR4. J. Neurochem. 95:544–552. 10.1111/j.1471-4159.2005.03410.x 16190873

[bib119] Oblander, S.A., and S.M. Brady-Kalnay. 2010. Distinct PTPmu-associated signaling molecules differentially regulate neurite outgrowth on E-, N-, and R-cadherin. Mol. Cell. Neurosci. 44:78–93. 10.1016/j.mcn.2010.02.005 20197094 PMC2881835

[bib120] Okuyama, Y., H. Nagashima, M. Ushio-Fukai, M. Croft, N. Ishii, and T. So. 2020. IQGAP1 restrains T-cell cosignaling mediated by OX40. FASEB J. 34:540–554. 10.1096/fj.201900879RR 31914585

[bib121] Osman, M.A., W.J. Antonisamy, and E. Yakirevich. 2020. IQGAP1 control of centrosome function defines distinct variants of triple negative breast cancer. Oncotarget. 11:2493–2511. 10.18632/oncotarget.27623 32655836 PMC7335670

[bib122] Pawson, T. 2002. Regulation and targets of receptor tyrosine kinases. Eur. J. Cancer. 38:S3–S10. 10.1016/S0959-8049(02)80597-4 12528767

[bib123] Peng, X., T. Wang, H. Gao, X. Yue, W. Bian, J. Mei, and Y. Zhang. 2021. The interplay between IQGAP1 and small GTPases in cancer metastasis. Biomed. Pharmacother. 135:111243. 10.1016/j.biopha.2021.111243 33434854

[bib124] Phillips-Mason, P.J., T.J. Gates, D.L. Major, D.B. Sacks, and S.M. Brady-Kalnay. 2006. The receptor protein-tyrosine phosphatase PTPmu interacts with IQGAP1. J. Biol. Chem. 281:4903–4910. 10.1074/jbc.M506414200 16380380

[bib125] Quinn, N.P., L. García-Gutiérrez, C. Doherty, A. von Kriegsheim, E. Fallahi, D.B. Sacks, and D. Matallanas. 2021. IQGAP1 is a scaffold of the core proteins of the Hippo pathway and negatively regulates the pro-apoptotic signal mediated by this pathway. Cells. 10:478. 10.3390/cells10020478 33672268 PMC7926663

[bib126] Ren, J.-G., Z. Li, D.L. Crimmins, and D.B. Sacks. 2005. Self-association of IQGAP1: Characterization and functional sequelae. J. Biol. Chem. 280:34548–34557. 10.1074/jbc.M507321200 16105843

[bib127] Ren, J.-G., Z. Li, and D.B. Sacks. 2007. IQGAP1 modulates activation of B-Raf. Proc. Natl. Acad. Sci. USA. 104:10465–10469. 10.1073/pnas.0611308104 17563371 PMC1965536

[bib128] Rigothier, C., P. Auguste, G.I. Welsh, S. Lepreux, C. Deminière, P.W. Mathieson, M.A. Saleem, J. Ripoche, and C. Combe. 2012. IQGAP1 interacts with components of the slit diaphragm complex in podocytes and is involved in podocyte migration and permeability in vitro. PLoS One. 7:e37695. 10.1371/journal.pone.0037695 22662192 PMC3360763

[bib129] Rittmeyer, E.N., S. Daniel, S.-C. Hsu, and M.A. Osman. 2008. A dual role for IQGAP1 in regulating exocytosis. J. Cell Sci. 121:391–403. 10.1242/jcs.016881 18216334

[bib130] Roy, M., Z. Li, and D.B. Sacks. 2004. IQGAP1 binds ERK2 and modulates its activity. J. Biol. Chem. 279:17329–17337. 10.1074/jbc.M308405200 14970219

[bib131] Roy, M., Z. Li, and D.B. Sacks. 2005. IQGAP1 is a scaffold for mitogen-activated protein kinase signaling. Mol. Cell. Biol. 25:7940–7952. 10.1128/MCB.25.18.7940-7952.2005 16135787 PMC1234344

[bib132] Ruiz-Velasco, R., C.C. Lanning, and C.L. Williams. 2002. The activation of Rac1 by M3 muscarinic acetylcholine receptors involves the translocation of Rac1 and IQGAP1 to cell junctions and changes in the composition of protein complexes containing Rac1, IQGAP1, and actin. J. Biol. Chem. 277:33081–33091. 10.1074/jbc.M202664200 12070151

[bib133] Saltiel, A.R. 2021. Insulin signaling in health and disease. J. Clin. Invest. 131:e142241. 10.1172/JCI142241 33393497 PMC7773347

[bib134] Salvi, R., C. Kumar, K. Brahmbhatt, R. Subedi, S. Idicula-Thomas, T. Madan, and B. Biswas. 2022. N-linked glycosylation in Chinese hamster ovary cells is critical for insulin-like growth factor 1 signaling. Int. J. Mol. Sci. 23:14952. 10.3390/ijms232314952 36499281 PMC9735751

[bib135] Sayedyahossein, S., Z. Li, A.C. Hedman, C.J. Morgan, and D.B. Sacks. 2016. IQGAP1 binds to yes-associated protein (YAP) and modulates its transcriptional activity. J. Biol. Chem. 291:19261–19273. 10.1074/jbc.M116.732529 27440047 PMC5016668

[bib136] Sbroggiò, M., D. Carnevale, A. Bertero, G. Cifelli, E. De Blasio, G. Mascio, E. Hirsch, W.F. Bahou, E. Turco, L. Silengo, . 2011. IQGAP1 regulates ERK1/2 and AKT signalling in the heart and sustains functional remodelling upon pressure overload. Cardiovasc. Res. 91:456–464. 10.1093/cvr/cvr103 21493702 PMC3294280

[bib137] Schmidt, V.A., L. Scudder, C.E. Devoe, A. Bernards, L.D. Cupit, and W.F. Bahou. 2003. IQGAP2 functions as a GTP-dependent effector protein in thrombin-induced platelet cytoskeletal reorganization. Blood. 101:3021–3028. 10.1182/blood-2002-09-2807 12515716

[bib138] Schrick, C., A. Fischer, D.P. Srivastava, N.C. Tronson, P. Penzes, and J. Radulovic. 2007. N-cadherin regulates cytoskeletally associated IQGAP1/ERK signaling and memory formation. Neuron. 55:786–798. 10.1016/j.neuron.2007.07.034 17785185 PMC2064867

[bib139] Sharma, S., G.M. Findlay, H.S. Bandukwala, S. Oberdoerffer, B. Baust, Z. Li, V. Schmidt, P.G. Hogan, D.B. Sacks, and A. Rao. 2011. Dephosphorylation of the nuclear factor of activated T cells (NFAT) transcription factor is regulated by an RNA-protein scaffold complex. Proc. Natl. Acad. Sci. USA. 108:11381–11386. 10.1073/pnas.1019711108 21709260 PMC3136327

[bib140] Sheen, Y.-S., M.-H. Lin, W.-C. Tzeng, and C.-Y. Chu. 2020. Purpuric drug eruptions induced by EGFR tyrosine kinase inhibitors are associated with IQGAP1-mediated increase in vascular permeability. J. Pathol. 250:452–463. 10.1002/path.5393 32030757

[bib141] Shi, Y., N. Qin, Q. Zhou, Y. Chen, S. Huang, B. Chen, G. Shen, and H. Jia. 2017. Role of IQGAP3 in metastasis and epithelial-mesenchymal transition in human hepatocellular carcinoma. J. Transl. Med. 15:176. 10.1186/s12967-017-1275-8 28810875 PMC5558666

[bib142] Shimao, Y., K. Nabeshima, T. Inoue, and M. Koono. 2002. Complex formation of IQGAP1 with E-cadherin/catenin during cohort migration of carcinoma cells. Its possible association with localized release from cell-cell adhesion. Virchows Arch. 441:124–132. 10.1007/s00428-002-0603-3 12189501

[bib143] Skandalis, S.S., I. Kozlova, U. Engström, U. Hellman, and P. Heldin. 2010. Proteomic identification of CD44 interacting proteins. IUBMB Life. 62:833–840. 10.1002/iub.392 21117172

[bib144] Smith, M.J., W.R. Hardy, G.-Y. Li, M. Goudreault, S. Hersch, P. Metalnikov, A. Starostine, T. Pawson, and M. Ikura. 2010. The PTB domain of ShcA couples receptor activation to the cytoskeletal regulator IQGAP1. EMBO J. 29:884–896. 10.1038/emboj.2009.399 20075861 PMC2837165

[bib145] Smith, J.M., A.C. Hedman, and D.B. Sacks. 2015. IQGAPs choreograph cellular signaling from the membrane to the nucleus. Trends Cell Biol. 25:171–184. 10.1016/j.tcb.2014.12.005 25618329 PMC4344846

[bib146] Smith, J.S., R.J. Lefkowitz, and S. Rajagopal. 2018. Biased signalling: From simple switches to allosteric microprocessors. Nat. Rev. Drug Discov. 17:243–260. 10.1038/nrd.2017.229 29302067 PMC5936084

[bib147] Suzuki, K., Y. Chikamatsu, and K. Takahashi. 2005. Requirement of protein phosphatase 2A for recruitment of IQGAP1 to Rac-bound β1 integrin. J. Cell. Physiol. 203:487–492. 10.1002/jcp.20249 15521075

[bib148] Takahashi, K., and K. Suzuki. 2006. Regulation of protein phosphatase 2A-mediated recruitment of IQGAP1 to β1 integrin by EGF through activation of Ca^2+^/calmodulin-dependent protein kinase II. J. Cell. Physiol. 208:213–219. 10.1002/jcp.20657 16557530

[bib149] Tanaka, M., and D.W. Siemann. 2020. Gas6/Axl signaling pathway in the tumor immune microenvironment. Cancers. 12:1850. 10.3390/cancers12071850 32660000 PMC7408754

[bib150] Tang, L.-Y., M. Heller, Z. Meng, L.-R. Yu, Y. Tang, M. Zhou, and Y.E. Zhang. 2017. Transforming growth factor-β (TGF-β) directly activates the JAK1-STAT3 axis to induce hepatic fibrosis in coordination with the SMAD pathway. J. Biol. Chem. 292:4302–4312. 10.1074/jbc.M116.773085 28154170 PMC5354477

[bib151] Tekletsadik, Y.K., R. Sonn, and M.A. Osman. 2012. A conserved role of IQGAP1 in regulating TOR complex 1. J. Cell Sci. 125:2041–2052. 10.1242/jcs.098947 22328503 PMC3360921

[bib152] Thines, L., Z. Li, and D.B. Sacks. 2023. IQGAP1 is a phosphotyrosine-regulated scaffold for SH2-containing proteins. Cells. 12:483. 10.3390/cells12030483 36766826 PMC9913818

[bib153] Tian, Y., X. Tian, G. Gawlak, J.J. O’Donnell III, D.B. Sacks, and A.A. Birukova. 2014. IQGAP1 regulates endothelial barrier function via EB1-cortactin cross talk. Mol. Cell. Biol. 34:3546–3558. 10.1128/MCB.00248-14 25022754 PMC4135611

[bib154] Tian, Y., G. Gawlak, A.S. Shah, K. Higginbotham, X. Tian, Y. Kawasaki, T. Akiyama, D.B. Sacks, and A.A. Birukova. 2015. Hepatocyte growth factor-induced Asef-IQGAP1 complex controls cytoskeletal remodeling and endothelial barrier. J. Biol. Chem. 290:4097–4109. 10.1074/jbc.M114.620377 25492863 PMC4326819

[bib155] Tonks, N.K. 2006. Protein tyrosine phosphatases: From genes, to function, to disease. Nat. Rev. Mol. Cell Biol. 7:833–846. 10.1038/nrm2039 17057753

[bib156] Vander Ark, A., J. Cao, and X. Li. 2018. TGF-β receptors: In and beyond TGF-β signaling. Cell. Signal. 52:112–120. 10.1016/j.cellsig.2018.09.002 30184463

[bib157] Vandercappellen, J., J. Van Damme, and S. Struyf. 2008. The role of CXC chemokines and their receptors in cancer. Cancer Lett. 267:226–244. 10.1016/j.canlet.2008.04.050 18579287

[bib158] Wang, S., T. Watanabe, J. Noritake, M. Fukata, T. Yoshimura, N. Itoh, T. Harada, M. Nakagawa, Y. Matsuura, N. Arimura, and K. Kaibuchi. 2007. IQGAP3, a novel effector of Rac1 and Cdc42, regulates neurite outgrowth. J. Cell Sci. 120:567–577. 10.1242/jcs.03356 17244649

[bib159] Wang, Z., M. Cai, L.W.R. Tay, F. Zhang, P. Wu, A. Huynh, X. Cao, G. Di Paolo, J. Peng, D.M. Milewicz, and G. Du. 2019. Phosphatidic acid generated by PLD2 promotes the plasma membrane recruitment of IQGAP1 and neointima formation. FASEB J. 33:6713–6725. 10.1096/fj.201800390RR 30811216 PMC6529346

[bib160] Wang, H., A. Ramshekar, E. Kunz, D.B. Sacks, and M.E. Hartnett. 2020. IQGAP1 causes choroidal neovascularization by sustaining VEGFR2-mediated Rac1 activation. Angiogenesis. 23:685–698. 10.1007/s10456-020-09740-y 32783108 PMC7530064

[bib161] Watanabe, T., S. Wang, J. Noritake, K. Sato, M. Fukata, M. Takefuji, M. Nakagawa, N. Izumi, T. Akiyama, and K. Kaibuchi. 2004. Interaction with IQGAP1 links APC to Rac1, Cdc42, and actin filaments during cell polarization and migration. Dev. Cell. 7:871–883. 10.1016/j.devcel.2004.10.017 15572129

[bib162] Wei, T., and P.F. Lambert. 2021. Role of IQGAP1 in carcinogenesis. Cancers. 13:3940. 10.3390/cancers13163940 34439095 PMC8391515

[bib163] Wei, T., S. Choi, D. Buehler, R.A. Anderson, and P.F. Lambert. 2020. A PI3K/AKT scaffolding protein, IQ motif–containing GTPase associating protein 1 (IQGAP1), promotes head and neck carcinogenesis. Clin. Cancer Res. 26:301–311. 10.1158/1078-0432.CCR-19-1063 31597661 PMC6942630

[bib164] Weis, W.I., and B.K. Kobilka. 2018. The molecular basis of G protein-coupled receptor activation. Annu. Rev. Biochem. 87:897–919. 10.1146/annurev-biochem-060614-033910 29925258 PMC6535337

[bib165] Weisshaar, N., J. Wu, Y. Ming, A. Madi, A. Hotz-Wagenblatt, S. Ma, A. Mieg, M. Hering, F. Zettl, K. Mohr, . 2022. Rgs16 promotes antitumor CD8+ T cell exhaustion. Sci. Immunol. 7:eabh1873. 10.1126/sciimmunol.abh1873 35622904

[bib166] White, C.D., M.D. Brown, and D.B. Sacks. 2009. IQGAPs in cancer: A family of scaffold proteins underlying tumorigenesis. FEBS Lett. 583:1817–1824. 10.1016/j.febslet.2009.05.007 19433088 PMC2743239

[bib167] White, C.D., Z. Li, D.A. Dillon, and D.B. Sacks. 2011. IQGAP1 protein binds human epidermal growth factor receptor 2 (HER2) and modulates trastuzumab resistance. J. Biol. Chem. 286:29734–29747. 10.1074/jbc.M111.220939 21724847 PMC3191015

[bib168] Wium, M., A.F. Ajayi-Smith, J.D. Paccez, and L.F. Zerbini. 2021. The role of the receptor tyrosine kinase Axl in carcinogenesis and development of therapeutic resistance: An overview of molecular mechanisms and future applications. Cancers. 13:1521. 10.3390/cancers13071521 33806258 PMC8037968

[bib169] Wolf, K.J., P. Shukla, K. Springer, S. Lee, J.D. Coombes, C.J. Choy, S.J. Kenny, K. Xu, and S. Kumar. 2020. A mode of cell adhesion and migration facilitated by CD44-dependent microtentacles. Proc. Natl. Acad. Sci. USA. 117:11432–11443. 10.1073/pnas.1914294117 32381732 PMC7261014

[bib170] Wootten, D., A. Christopoulos, M. Marti-Solano, M.M. Babu, and P.M. Sexton. 2018. Mechanisms of signalling and biased agonism in G protein-coupled receptors. Nat. Rev. Mol. Cell Biol. 19:638–653. 10.1038/s41580-018-0049-3 30104700

[bib171] Wu, Y., Y.-C. Chen, J.-R. Sang, and W.-R. Xu. 2011. RhoC protein stimulates migration of gastric cancer cells through interaction with scaffold protein IQGAP1. Mol. Med. Rep. 4:697–703. 10.3892/mmr.2011.482 21537845

[bib172] Xie, L., B.K. Law, M.E. Aakre, M. Edgerton, Y. Shyr, N.A. Bhowmick, and H.L. Moses. 2003. Transforming growth factor beta-regulated gene expression in a mouse mammary gland epithelial cell line. Breast Cancer Res. 5:R187–R198. 10.1186/bcr640 14580254 PMC314403

[bib173] Yamaoka-Tojo, M., M. Ushio-Fukai, L. Hilenski, S.I. Dikalov, Y.E. Chen, T. Tojo, T. Fukai, M. Fujimoto, N.A. Patrushev, N. Wang, . 2004. IQGAP1, a novel vascular endothelial growth factor receptor binding protein, is involved in reactive oxygen species--dependent endothelial migration and proliferation. Circ. Res. 95:276–283. 10.1161/01.RES.0000136522.58649.60 15217908

[bib174] Yamaoka-Tojo, M., T. Tojo, H.W. Kim, L. Hilenski, N.A. Patrushev, L. Zhang, T. Fukai, and M. Ushio-Fukai. 2006. IQGAP1 mediates VE-cadherin-based cell-cell contacts and VEGF signaling at adherence junctions linked to angiogenesis. Arterioscler. Thromb. Vasc. Biol. 26:1991–1997. 10.1161/01.ATV.0000231524.14873.e7 16763158

[bib175] Yang, Y., W. Zhao, Q.-W. Xu, X.-S. Wang, Y. Zhang, and J. Zhang. 2014. IQGAP3 promotes EGFR-ERK signaling and the growth and metastasis of lung cancer cells. PLoS One. 9:e97578. 10.1371/journal.pone.0097578 24849319 PMC4029748

[bib176] Yarden, Y. 2001. Biology of HER2 and its importance in breast cancer. Oncology. 61:1–13. 10.1159/000055396 11694782

[bib177] Yu, W., L. Yang, T. Li, and Y. Zhang. 2019. Cadherin signaling in cancer: Its functions and role as a therapeutic target. Front. Oncol. 9:989. 10.3389/fonc.2019.00989 31637214 PMC6788064

[bib178] Yuan, Z., W. Zhang, and W. Tan. 2013. A labile pool of IQGAP1 disassembles endothelial adherens junctions. Int. J. Mol. Sci. 14:13377–13390. 10.3390/ijms140713377 23807500 PMC3742192

[bib179] Zajac, M., J. Law, D.D. Cvetkovic, M. Pampillo, L. McColl, C. Pape, G.M. Di Guglielmo, L.M. Postovit, A.V. Babwah, and M. Bhattacharya. 2011. GPR54 (KISS1R) transactivates EGFR to promote breast cancer cell invasiveness. PLoS One. 6:e21599. 10.1371/journal.pone.0021599 21738726 PMC3125256

[bib180] Zheng, X., X. Hu, and W. Zhang. 2019a. The phenotype of vascular smooth muscle cells co-cultured with endothelial cells is modulated by PDGFR-β/IQGAP1 signaling in LPS-induced intravascular injury. Int. J. Med. Sci. 16:1149–1156. 10.7150/ijms.34749 31523178 PMC6743276

[bib181] Zheng, X., W. Zhang, and Z. Wang. 2019b. Simvastatin preparations promote PDGF-BB secretion to repair LPS-induced endothelial injury through the PDGFRβ/PI3K/Akt/IQGAP1 signalling pathway. J. Cell. Mol. Med. 23:8314–8327. 10.1111/jcmm.14709 31576676 PMC6850957

[bib182] Zong, C., X. Zhang, Y. Xie, and J. Cheng. 2015. Transforming growth factor-β inhibits IQ motif containing guanosine triphosphatase activating protein 1 expression in lung fibroblasts via the nuclear factor-κB signaling pathway. Mol. Med. Rep. 12:442–448. 10.3892/mmr.2015.3353 25684348

